# Early prone positioning in acute respiratory distress syndrome related to COVID-19: a propensity score analysis from the multicentric cohort COVID-ICU network—the ProneCOVID study

**DOI:** 10.1186/s13054-022-03949-7

**Published:** 2022-03-24

**Authors:** Christophe Le Terrier, Florian Sigaud, Said Lebbah, Luc Desmedt, David Hajage, Claude Guérin, Jérôme Pugin, Steve Primmaz, Nicolas Terzi, COVID-ICU Group on behalf of the REVA Network and the COVID-ICU Investigators, Alain Mercat, Pierre Asfar, François Beloncle, Julien Demiselle, Tài Pham, Arthur Pavot, Xavier Monnet, Christian Richard, Alexandre Demoule, Martin Dres, Julien Mayaux, Alexandra Beurton, Cédric Daubin, Richard Descamps, Aurélie Joret, Damien Du Cheyron, Frédéric Pene, Jean-Daniel Chiche, Mathieu Jozwiak, Paul Jaubert, Guillaume Voiriot, Muriel Fartoukh, Marion Teulier, Clarisse Blayau, Erwen L’Her, Cécile Aubron, Laetitia Bodenes, Nicolas Ferriere, Johann Auchabie, Anthony Le Meur, Sylvain Pignal, Thierry Mazzoni, Jean-Pierre Quenot, Pascal Andreu, Jean-Baptiste Roudau, Marie Labruyère, Saad Nseir, Sébastien Preau, Julien Poissy, Daniel Mathieu, Sarah Benhamida, Rémi Paulet, Nicolas Roucaud, Martial Thyrault, Florence Daviet, Sami Hraiech, Gabriel Parzy, Aude Sylvestre, Sébastien Jochmans, Anne-Laure Bouilland, Mehran Monchi, Marc Danguy des Déserts, Quentin Mathais, Gwendoline Rager, Pierre Pasquier, Reignier Jean, Seguin Amélie, Garret Charlotte, Canet Emmanuel, Jean Dellamonica, Clément Saccheri, Romain Lombardi, Yanis Kouchit, Sophie Jacquier, Armelle Mathonnet, Mai-Ahn Nay, Isabelle Runge, Frédéric Martino, Laure Flurin, Amélie Rolle, Michel Carles, Rémi Coudroy, Arnaud W. Thille, Jean-Pierre Frat, Maeva Rodriguez, Pascal Beuret, Audrey Tientcheu, Arthur Vincent, Florian Michelin, Fabienne Tamion, Dorothée Carpentier, Déborah Boyer, Christophe Girault, Valérie Gissot, Stéphan Ehrmann, Charlotte Salmon Gandonniere, Djlali Elaroussi, Agathe Delbove, Yannick Fedun, Julien Huntzinger, Eddy Lebas, Grâce Kisoka, Céline Grégoire, Stella Marchetta, Bernard Lambermont, Laurent Argaud, Thomas Baudry, Pierre-Jean Bertrand, Auguste Dargent, Christophe Guitton, Nicolas Chudeau, Mickaël Landais, Cédric Darreau, Alexis Ferre, Antoine Gros, Guillaume Lacave, Fabrice Bruneel, Mathilde Neuville, Jérôme Devaquet, Guillaume Tachon, Richard Gallot, Riad Chelha, Arnaud Galbois, Anne Jallot, Ludivine Chalumeau Lemoine, Khaldoun Kuteifan, Valentin Pointurier, Louise-Marie Jandeaux, Joy Mootien, Charles Damoisel, Benjamin Sztrymf, Matthieu Schmidt, Alain Combes, Juliette Chommeloux, Charles Edouard Luyt, Frédérique Schortgen, Leon Rusel, Camille Jung, Florent Gobert, Damien Vimpere, Lionel Lamhaut, Bertrand Sauneuf, Liliane Charrrier, Julien Calus, Isabelle Desmeules, Benoît Painvin, Jean-Marc Tadie, Vincent Castelain, Baptiste Michard, Jean-Etienne Herbrecht, Mathieu Baldacini, Nicolas Weiss, Sophie Demeret, Clémence Marois, Benjamin Rohaut, Pierre-Henri Moury, Anne-Charlotte Savida, Emmanuel Couadau, Mathieu Série, Nica Alexandru, Cédric Bruel, Candice Fontaine, Sonia Garrigou, Juliette Courtiade Mahler, Maxime Leclerc, Michel Ramakers, Pierre Garçon, Nicole Massou, Ly Van Vong, Juliane Sen, Nolwenn Lucas, Franck Chemouni, Annabelle Stoclin, Alexandre Avenel, Henri Faure, Angélie Gentilhomme, Sylvie Ricome, Paul Abraham, Céline Monard, Julien Textoris, Thomas Rimmele, Florent Montini, Gabriel Lejour, Thierry Lazard, Isabelle Etienney, Younes Kerroumi, Claire Dupuis, Marine Bereiziat, Elisabeth Coupez, François Thouy, Clément Hoffmann, Nicolas Donat, Anne Chrisment, Rose-Marie Blot, Antoine Kimmoun, Audrey Jacquot, Matthieu Mattei, Bruno Levy, Ramin Ravan, Loïc Dopeux, Jean-Mathias Liteaudon, Delphine Roux, Brice Rey, Radu Anghel, Deborah Schenesse, Vincent Gevrey, Jermy Castanera, Philippe Petua, Benjamin Madeux, Otto Hartman, Michael Piagnerelli, Anne Joosten, Cinderella Noel, Patrick Biston, Thibaut Noel, Gurvan L. E. Bouar, Messabi Boukhanza, Elsa Demarest, Marie-France Bajolet, Nathanaël Charrier, Audrey Quenet, Cécile Zylberfajn, Nicolas Dufour, Buno Mégarbane, Sqébastian Voicu, Nicolas Deye, Isabelle Malissin, François Legay, Matthieu Debarre, Nicolas Barbarot, Pierre Fillatre, Bertrand Delord, Thomas Laterrade, Tahar Saghi, Wilfried Pujol, Pierre Julien Cungi, Pierre Esnault, Mickael Cardinale, Vivien Hong Tuan Ha, Grégory Fleury, Marie-Ange Brou, Daniel Zafimahazo, David Tran-Van, Patrick Avargues, Lisa Carenco, Nicolas Robin, Alexandre Ouali, Lucie Houdou, Christophe Le Terrier, Noémie Suh, Steve Primmaz, Jérôme Pugin, Emmanuel Weiss, Tobias Gauss, Jean-Denis Moyer, Catherine Paugam Burtz, Béatrice La Combe, Rolland Smonig, Jade Violleau, Pauline Cailliez, Jonathan Chelly, Antoine Marchalot, Cécile Saladin, Christelle Bigot, Pierre-Marie Fayolle, Jules Fatséas, Amr Ibrahim, Dabor Resiere, Rabih Hage, Clémentine Cholet, Marie Cantier, Pierre Trouiler, Philippe Montravers, Brice Lortat-Jacob, Sebastien Tanaka, Alexy Tran Dinh, Jacques Duranteau, Anatole Harrois, Guillaume Dubreuil, Marie Werner, Anne Godier, Sophie Hamada, Diane Zlotnik, Hélène Nougue, Armand Mekontso-Dessap, Guillaume Carteaux, Keyvan Razazi, Nicolas De Prost, Nicolas Mongardon, Olivier Langeron, Eric Levesque, Arié Attias, Charles de Roquetaillade, Benjamin G. Chousterman, Alexandre Mebazaa, Etienne Gayat, Marc Garnier, Emmanuel Pardo, Lea Satre-Buisson, Christophe Gutton, Elise Yvin, Clémence Marcault, Elie Azoulay, Michael Darmon, Hafid Ait Oufella, Geoffroy Hariri, Tomas Urbina, Sandie Mazerand, Nicholas Heming, Francesca Santi, Pierre Moine, Djillali Annane, Adrien Bouglé, Edris Omar, Aymeric Lancelot, Emmanuelle Begot, Gaétan Plantefeve, Damien Contou, Hervé Mentec, Olivier Pajot, Stanislas Faguer, Olivier Cointault, Laurence Lavayssiere, Marie-Béatrice Nogier, Matthieu Jamme, Claire Pichereau, Jan Hayon, Hervé Outin, François Dépret, Maxime Coutrot, Maité Chaussard, Lucie Guillemet, Pierre Goffin, Romain Thouny, Julien Guntz, Laurent Jadot, Romain Persichini, Vanessa Jean-Michel, Hugues Georges, Thomas Caulier, Gaël Pradel, Marie-Hélène Hausermann, Thi My Hue Nguyen-Valat, Michel Boudinaud, Emmanuel Vivier, Sylvène Rosseli, Gaël Bourdin, Christian Pommier, Marc Vinclair, Simon Poignant, Sandrine Mons, Wulfran Bougouin, Franklin Bruna, Quentin Maestraggi, Christian Roth, Laurent Bitker, François Dhelft, Justine Bonnet-Chateau, Mathilde Filippelli, Tristan Morichau-Beauchant, Stéphane Thierry, Charlotte Le Roy, Mélanie Saint Jouan, Bruno Goncalves, Aurélien Mazeraud, Matthieu Daniel, Tarek Sharshar, Cyril Cadoz, Rostane Gaci, Sébastien Gette, Guillaune Louis, Sophe-Caroline Sacleux, Marie-Amélie Ordan, Aurélie Cravoisy, Marie Conrad, Guilhem Courte, Sébastien Gibot, Younès Benzidi, Claudia Casella, Laurent Serpin, Jean-Lou Setti, Marie-Catherine Besse, Anna Bourreau, Jérôme Pillot, Caroline Rivera, Camille Vinclair, Marie-Aline Robaux, Chloé Achino, Marie-Charlotte Delignette, Tessa Mazard, Frédéric Aubrun, Bruno Bouchet, Aurélien Frérou, Laura Muller, Charlotte Quentin, Samuel Degoul, Xavier Stihle, Claude Sumian, Nicoletta Bergero, Bernard Lanaspre, Hervé Quintard, Eve Marie Maiziere, Pierre-Yves Egreteau, Guillaume Leloup, Florin Berteau, Marjolaine Cottrel, Marie Bouteloup, Matthieu Jeannot, Quentin Blanc, Julien Saison, Isabelle Geneau, Romaric Grenot, Abdel Ouchike, Pascal Hazera, Anne-Lyse Masse, Suela Demiri, Corinne Vezinet, Elodie Baron, Deborah Benchetrit, Antoine Monsel, Grégoire Trebbia, Emmanuelle Schaack, Raphaël Lepecq, Mathieu Bobet, Christophe Vinsonneau, Thibault Dekeyser, Quentin Delforge, Imen Rahmani, Bérengère Vivet, Jonathan Paillot, Lucie Hierle, Claire Chaignat, Sarah Valette, Benoït Her, Jennifier Brunet, Mathieu Page, Fabienne Boiste, Anthony Collin, Florent Bavozet, Aude Garin, Mohamed Dlala, Kais Mhamdi, Bassem Beilouny, Alexandra Lavalard, Severine Perez, Benoit Veber, Pierre-Gildas Guitard, Philippe Gouin, Anna Lamacz, Fabienne Plouvier, Bertrand P. Delaborde, Aïssa Kherchache, Amina Chaalal, Jean-Damien Ricard, Marc Amouretti, Santiago Freita-Ramos, Damien Roux, Jean-Michel Constantin, Mona Assefi, Marine Lecore, Agathe Selves, Florian Prevost, Christian Lamer, Ruiying Shi, Lyes Knani, Sébastien Pili Floury, Lucie Vettoretti, Michael Levy, Lucile Marsac, Stéphane Dauger, Sophie Guilmin-Crépon, Hadrien Winiszewski, Gael Piton, Thibaud Soumagne, Gilles Capellier, Jean-Baptiste Putegnat, Frédérique Bayle, Maya Perrou, Ghyslaine Thao, Guillaume Géri, Cyril Charron, Xavier Repessé, Antoine Vieillard-Baron, Mathieu Guilbart, Pierre-Alexandre Roger, Sébastien Hinard, Pierre-Yves Macq, Kevin Chaulier, Sylvie Goutte, Patrick Chillet, Anaïs Pitta, Barbara Darjent, Amandine Bruneau, Sigismond Lasocki, Maxime Leger, Soizic Gergaud, Pierre Lemarie, Nicolas Terzi, Carole Schwebel, Anaïs Dartevel, Louis-Marie Galerneau, Jean-Luc Diehl, Caroline Hauw-Berlemont, Nicolas Péron, Emmanuel Guérot, Abolfazl Mohebbi Amoli, Michel Benhamou, Jean-Pierre Deyme, Olivier Andremont, Diane Lena, Julien Cady, Arnaud Causeret, Arnaud De La Chapelle, Christophe Cracco, Stéphane Rouleau, David Schnell, Camille Foucault, Cécile Lory, Thibault Chapelle, Vincent Bruckert, Julie Garcia, Abdlazize Sahraoui, Nathalie Abbosh, Caroline Bornstain, Pierre Pernet, Florent Poirson, Ahmed Pasem, Philippe Karoubi, Virginie Poupinel, Caroline Gauthier, François Bouniol, Philippe Feuchere, Florent Bavozet, Anne Heron, Serge Carreira, Malo Emery, Anne Sophie Le Floch, Luana Giovannangeli, Nicolas Herzog, Christophe Giacardi, Thibaut Baudic, Chloé Thill, Said Lebbah, Jessica Palmyre, Florence Tubach, David Hajage, Nicolas Bonnet, Nathan Ebstein, Stéphane Gaudry, Yves Cohen, Julie Noublanche, Olivier Lesieur, Arnaud Sément, Isabel Roca-Cerezo, Michel Pascal, Nesrine Sma, Gwenhaël Colin, Jean-Claude Lacherade, Gauthier Bionz, Natacha Maquigneau, Pierre Bouzat, Michel Durand, Marie-Christine Hérault, Jean-Francois Payen

**Affiliations:** 1 Division of Intensive Care, Geneva University Hospitals and the University of Geneva Faculty of Medicine, Geneva, Switzerland; 2 Medical Intensive Care Unit, Grenoble Alpes University Hospital, Grenoble, France; 3 AP-HP, Département de Santé Publique, Centre de Pharmaco-épidémiologie, Paris, France; 4 Medical Intensive Care Unit, Nantes Hôtel-Dieu University Hospital, Nantes, France; 5 Division of Intensive Care, Edouard Herriot University Hospital, Lyon, France; 6 Medical Intensive Care Unit, Grenoble Alpes University Hospital, Avenue Maquis du Grésivaudan, 38700 La Tronche, France

**Keywords:** Acute respiratory distress syndrome, Intubation, COVID-19, Mortality, Prone position, Intensive care unit

## Abstract

**Background:**

Delaying time to prone positioning (PP) may be associated with higher mortality in acute respiratory distress syndrome (ARDS) due to coronavirus disease 2019 (COVID-19). We evaluated the use and the impact of early PP on clinical outcomes in intubated patients hospitalized in intensive care units (ICUs) for COVID-19.

**Methods:**

All intubated patients with ARDS due to COVID-19 were involved in a secondary analysis from a prospective multicenter cohort study of COVID-ICU network including 149 ICUs across France, Belgium and Switzerland. Patients were followed-up until Day-90. The primary outcome was survival at Day-60. Analysis used a Cox proportional hazard model including a propensity score.

**Results:**

Among 2137 intubated patients, 1504 (70.4%) were placed in PP during their ICU stay and 491 (23%) during the first 24 h following ICU admission. One hundred and eighty-one patients (36.9%) of the early PP group had a PaO_2_/FiO_2_ ratio > 150 mmHg when prone positioning was initiated. Among non-early PP group patients, 1013 (47.4%) patients had finally been placed in PP within a median delay of 3 days after ICU admission. Day-60 mortality in non-early PP group was 34.2% versus 39.3% in the early PP group (*p* = 0.038). Day-28 and Day-90 mortality as well as the need for adjunctive therapies was more important in patients with early PP. After propensity score adjustment, no significant difference in survival at Day-60 was found between the two study groups (HR 1.34 [0.96–1.68], *p* = 0.09 and HR 1.19 [0.998–1.412], *p* = 0.053 in complete case analysis or in multiple imputation analysis, respectively).

**Conclusions:**

In a large multicentric international cohort of intubated ICU patients with ARDS due to COVID-19, PP has been used frequently as a main treatment. In this study, our data failed to show a survival benefit associated with early PP started within 24 h after ICU admission compared to PP after day-1 for all COVID-19 patients requiring invasive mechanical ventilation regardless of their severity.

**Supplementary Information:**

The online version contains supplementary material available at 10.1186/s13054-022-03949-7.

## Introduction

Since 2020, the world has been facing a global threat due to the COVID-19, overwhelming hospitals and intensive care units (ICUs) as never before. To date, the World Health Organization has reported 158 millions confirmed COVID-19 cases and more than 3 millions of deaths [1]. Patients infected by the severe acute respiratory syndrome coronavirus 2 (SARS-CoV-2) and hospitalized for a severe pneumonia may develop acute respiratory distress syndrome (ARDS), which is associated with high mortality [2–4]. Therefore, an extensive burden brought upon the intensive care units (ICUs) to provide invasive mechanical ventilation and other advanced forms of life support [5].

Before the COVID-19 pandemic, the Proseva trial [6] demonstrated an improvement in survival from prone position (PP) used as cycles of more than 16 consecutive hrs in selected ARDS patients, i.e., those with a PaO_2_/FIO_2_ ratio < 150 mmHg after 12 to 24 h-stabilization period. Though experts recommended PP in this setting [7], in the daily practice the rate of use of PP was lower than expected [8]. Since the beginning of the COVID-19 pandemic, the surviving sepsis campaign (SSC) recommended PP in COVID-19 presenting with ARDS [9], a treatment widely adopted even though the level of evidence was similar as before the pandemic [4, 10]. In this recommendation, no timing to start prone position was proposed. Owing to the very large number of COVID-19-related ARDS treated with PP it was reported that an early application of PP [11, 12] and the response to PP in terms of oxygenation [13, 14] were both possibly associated with a better outcome. Even if some studies of patients report interesting results [11–14], the impact of early PP on mortality remains unclear in COVID-19 patients in the ICU.

The objective of the present ancillary study was to analyze the use of early PP in the ICU management of ARDS patient due to COVID-19 and to evaluate the impact of an early PP on survival, as well as on respiratory system mechanics and oxygenation, using a large international cohort of COVID-19 ARDS patients [4].

## Methods

### Study design and patients

This study was a secondary analysis of the COVID-ICU study [4]. COVID-ICU was a prospective, multicenter observational cohort study of 149 ICUs from 138 hospitals conducted across three European countries (France, Belgium and Switzerland). The ethical committees of Switzerland (BASEC #: 2020-00704), of the French Intensive Care Society (CE-SRLF 20-23) and of Belgium (2020-294) approved this study and all patients or relatives had given their consent to be included in the COVID-ICU cohort. It recruited 4643 patients between February and May 2020 with 80% of patients receiving invasive mechanical ventilation during their ICU stay.

All consecutive patients over 16 year-old included from February 25, 2020, to May 4, 2020, in the COVID-ICU study with an available vital status at Day-90 were eligible. Patients who met the following criteria in the first 24 h after admission were included: intubated and mechanically ventilated, PaO_2_/FiO_2_ < 300 mmHg with PEEP > 5 cmH_2_O and no therapeutic limitations. Laboratory confirmation for SARS-CoV-2 was defined as a positive result of real-time reverse transcriptase-polymerase chain reaction (RT-PCR) assay from either nasal or pharyngeal swabs, and/or lower respiratory tract aspirates. Patients without laboratory-confirmed COVID-19 were not included, even if they presented with a typical radiological pattern.

Patients were classified according to the fact that they had been subjected to PP at Day-1 or later. Day-1 was defined as the first day in ICU at 10 am following the COVID-ICU study. All patients placed in PP during their first day in ICU constituted the early PP group. All patients placed in PP after Day-1 or non-placed in PP during their ICU stay were categorized in the non-early prone position group. Patients placed in PP later in their ICU course were included in the non-early proning group to reduce the potential for immortal time bias and to emulate an intention-to-treat strategy of a randomized trial. Indication for invasive mechanical ventilation and mechanical ventilation settings was left to the discretion of the participating centers.

### Data collection

A standardized electronic case report form was completed each day at 10 am by the study investigators. Baseline characteristics were collected at ICU admission: age, sex, body mass index (BMI), active smoking, Simplified Acute Physiology Score (SAPS) II score, Sequential Organ Failure Assessment (SOFA), treated hypertension, diabetes, long-term corticosteroids, immunodeficiency, Clinical Frailty Scale, the date of the first symptom and dates of the hospital and ICU admissions. All investigators were asked to provide the lowest arterial partial pressure of oxygen (PaO_2_) at Day-1 after intubation and the corresponding fraction of oxygen inspired (FiO_2_) to calculate PaO_2_/FiO_2_ ratio and categorized according to the ARDS Berlin definition [15]. Static compliance was defined by dividing the tidal volume by the driving pressure. The driving pressure was calculated by subtracting plateau pressure from positive end-expiratory pressure (PEEP). All biological data were collected at ICU admission. Proved concurrent bacterial pneumonia was defined by a positive bacterial culture at ICU admission in either a bronchoalveolar lavage sample, or in a blind protected specimen brush distal, or in endotracheal aspirates. The main outcome was Day-60 survival. Secondary outcomes included Day-28 and Day-90 mortality, ventilator-free days until Day-28, extracorporeal membrane oxygenation (ECMO) requirement, extracorporeal CO_2_ removal (ECCO_2_R) requirement and inhaled nitric oxide. The ventilator-free days were computed as the number of days that a patient was alive and free of invasive ventilation, calculated from ICU admission until Day-28. Patients who died before Day-28 or received invasive ventilation for more than 28 days were considered to have 0 ventilator-free days [16]. The static compliance, the SOFA score and the PaO_2_/FiO_2_ ratio were also evaluated at Day-3, Day-5 and Day-7 as secondary outcomes.

### Statistical analysis

Characteristics of patients were described as counts and percentages for categorical variables, and as mean and standard deviation or median and interquartile range for quantitative variables. Categorical variables were compared by Chi-square or Fisher's exact test, and quantitative variables were compared by Student's *t* test or Wilcoxon's rank-sum test. Kaplan–Meier overall survival curves until Day-28, Day-60 and Day-90 were computed.

The primary endpoint was the Day-60 survival according to prone positioning at Day-1 of ICU stay. To assess prone positioning at Day-1 effect on Day-60 survival, we used a Cox proportional hazard model weighted on inverse probability of treatment weighting (IPTW) using propensity score (PS) defined as the predictive probability of prone positioning conditional on measured baseline covariates [17]. The variables used to estimate propensity score were: age, gender, clinical frailty scale, SOFA cardiovascular, SOFA renal, SOFA coagulation, SAPS II score, immunodepression, long-term corticosteroids, treated hypertension, diabetes, BMI, delay between first symptoms and ICU admission, bacterial coinfection, ICU admission period (March 29 or after vs. March 28 or before), PaO_2_/FiO_2_ ratio and static compliance. A multivariate logistic regression model was performed to estimate the PS for each patient. To assess the balance of measured covariates between treatment groups, we used the standardized mean differences before and after PS weighting [18]. Then, a Cox proportional hazard model weighted on IPTW was performed to estimate the average treatment effect in the entire eligible population [17]. Hazard ratio and its 95% confidence interval were then estimated for the Day-60 mortality associated with prone positioning at Day-1. This analysis was performed on the complete cases data set, and a sensitivity analysis was performed using multiple imputations due to missing data. Imputation method, missing data were realized according to Vesin et al. [19]. Proportional hazard assumption was assessed by inspecting the scaled Schoenfeld residuals and Harrel’s test [20]. Multicollinearity was checked using variance inflation factor.

The secondary endpoints were: Day-28 survival, Day-90 survival, number of days free of mechanical ventilation up to Day-28, the need for extracorporeal life support, the need for inhaled nitric oxide, static compliance (at Day-3, 5 and 7), PaO_2_/FiO_2_ (at Day-3, 5 and 7) and SOFA score (Day-7, 21 and 28).

Subgroup analyses of mortality at Day-28, Day-60 and Day-90 were performed, according to PaO_2_/FiO_2_ at Day-1 (< or ≥ 150 mmHg) and time from ICU admission to the first prone position (< or ≥ 24 h). Subgroup analysis according to PaO_2_/FiO_2_ at Day-1 (< or ≥ 150 mmHg) also included a Cox proportional hazard model weighted on IPTW using propensity score to assess prone positioning at Day-1 effect on Day-60 survival.

All analyses were performed at a two-sided *α* level of 5% and conducted with R version 3.5.1 (R Foundation for Statistical Computing, Vienna, Austria).

## Results

### Characteristics of ICU patients

**Fig. 1:**
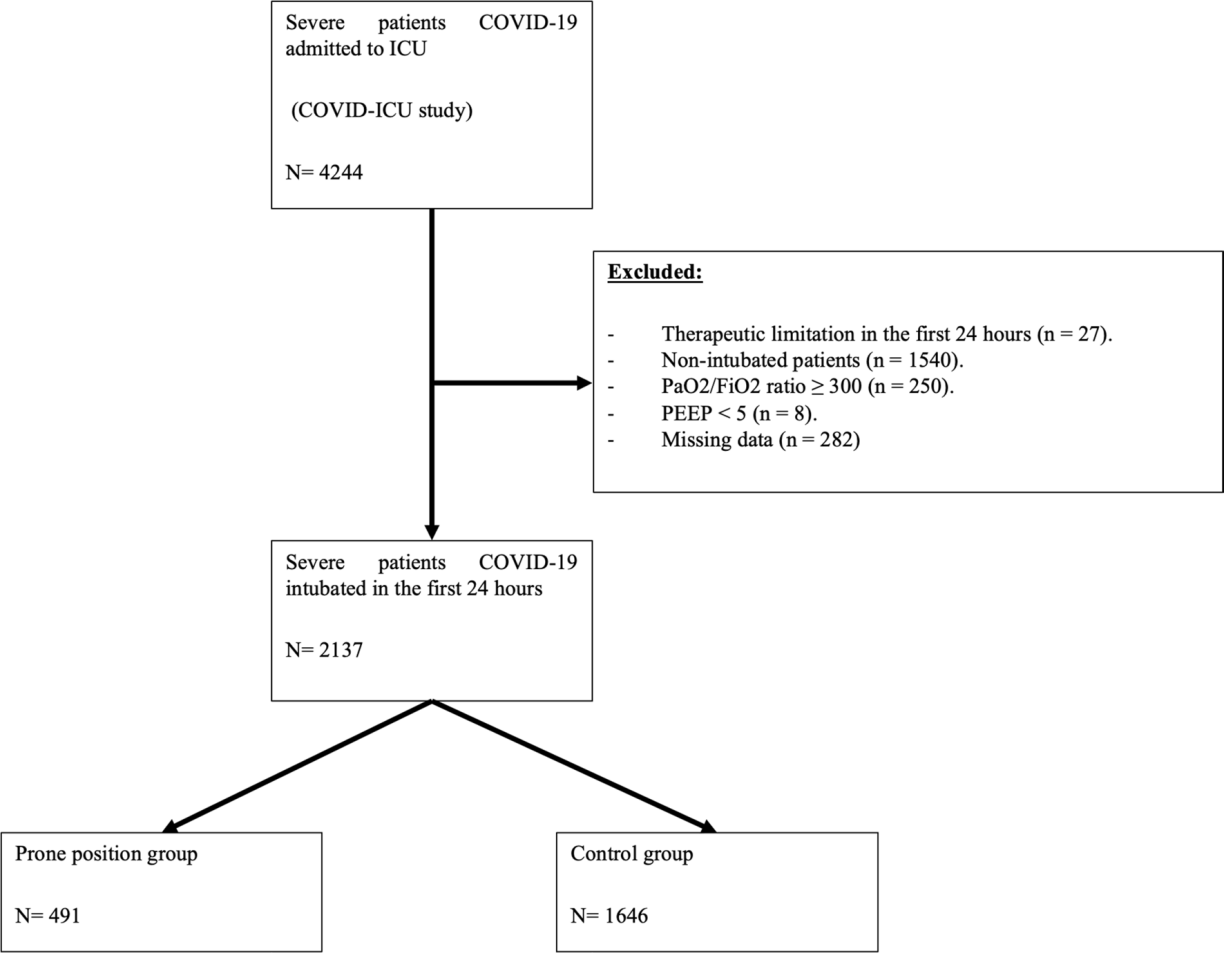
Study flowchart. *ICU* intensive care unit, *PaO*_*2*_ arterial partial pressure of oxygen, *FiO*_*2*_ fraction inspired of oxygen, *PEEP* positive end-expiratory pressure

COVID-ICU study enrolled 4244 patients. In this secondary analysis, 2137 patients met the inclusion criteria and were involved (Fig. 1). The median [interquartile range] age was 63 [55–70] years, 1598 (75.1%) of patients were male, with a median BMI of 29 [26–33] kg/m^2^. The median SAPS II, SOFA and Frailty score were 43 [32–56], 7 [4–10] and 2 [2–3] respectively. The main comorbidity was hypertension (49.9%), followed by diabetes (28.4%) and immunosuppression (7.3%). All patients were rapidly intubated after ICU admission with a median delay inferior to 3 h approximately. Regarding the ARDS severity at Day-1, the median static compliance was 32.8 [26.3–41.7] mL/cmH_2_O, and the PaO_2_/FiO_2_ ratio was 145.7 [101.7–200] mmHg including 1106 (51.8%) patients with a ratio less than 150 mmHg. All other baseline characteristics of patients are summarized in Table 1.

**Table 1:** Demographic, clinical and ventilatory characteristics of patients according to their proning status at Day-1

Variable	All patients (*n* = 2137)	Non-early prone position group (*n* = 1646)	Early prone position group (*n* = 491)	*p*
Age (years), median (IQR)	63 (55–70)	63 (55–70)	63 (54–70)	0.393
Sex, *n* (%)				
Men	1598 (75.1%)	1242 (75.7%)	356 (73.1%)	0.238
Women	529 (24.9%)	398 (24.3%)	131 (26.9%)	
Body mass index (kg/m^2^), median (IQR)	29 (26–33)	28 (26–32)	30 (27–34)	< 0.0001
≥ 30 kg/m^2^, *n* (%)	888 (44.4%)	636 (41.4%)	252 (54.8%)	< 0.0001
Comorbidities, *n* (%)				
Active smokers	87 (4.2%)	68 (4.3%)	19 (4%)	0.791
Treated hypertension	1055 (49.9%)	786 (48.3%)	269 (55.2%)	0.005
Known diabetes	601 (28.4%)	446 (27.4%)	155 (31.9%)	0.053
Immunodeficiency	154 (7.3%)	120 (7.4%)	34 (7%)	0.788
Long-term corticosteroids	77 (3.7%)	66 (4.1%)	11 (2.3%)	0.064
SAPS II score, median (IQR)	43 (32–56)	42 (32–56)	44 (32–55)	0.702
SOFA score at ICU admission, median (IQR)	7 (4–10)	7 (4–10)	8 (5–10)	0.033
Clinical frailty score, median (IQR)	2 (2–3)	2 (2–3)	2 (2–3)	0.112
Time between first symptoms and ICU admission (days), median (IQR)	9 (6–12)	9 (6–12)	9 (6–11)	0.273
Time between ICU admission and invasive mechanical ventilation (hours), median (IQR)	2.7 (0.7–9.7)	3 (0.7–10.8)	1.8 (0.4–6.3)	0.001
Concomitant bacterial pneumonia, *n* (%)	130 (6.3%)	93 (5.8%)	37 (7.7%)	0.143
Respiratory support received in ICU before intubation at Day-1, *n* (%)				
Oxygen therapy	220 (10.3%)	177 (10.8%)	43 (8.8%)	0.203
High-flow nasal cannula	143 (6.8%)	119 (7.3%)	24 (4.9%)	0.069
Non-invasive mechanical ventilation	61 (2.9%)	43 (2.3%)	18 (3.7%)	0.216
High-doses Corticosteroids using at Day-1 *n* (%)	227 (10.7%)	171 (10.5%)	56 (11.4%)	0.557
Invasive mechanical ventilation settings, median (IQR)				
PaO_2_/FiO_2_ (mmHg)	145.7 (101.7–200)	152.2 (107–205)	128.3 (87.5–177.5)	< 0.0001
Tidal volume (mL)	415 (375–450)	418 (380–450)	400 (370–440)	0.0035
Tidal volume, mL/kg PBW	6.1 (5.8–6.7)	6.1 (5.8–6.7)	6.1 (5.8–6.5)	0.1326
Set PEEP (cmH_2_O)	12 (10–14)	12 (10–14)	12 (10–14)	< 0.0001
Plateau pressure (cmH_2_O)	24 (21–27)	24 (21–27)	25 (22–28)	< 0.0001
Driving pressure^1^ (cmH_2_O)	13 (10–17)	13 (10–17)	13.5 (11–17)	0.0345
Mechanical power^2^ (J/min)	26.7 (18.9–35)	25.8 (18.4–33.6)	30.3 (21.1–39.1)	< 0.0001
Ventilatory ratio^3^ (J/min)	1.7 (1.4–2.2)	1.7 (1.4–2.1)	1.9 (1.5–2.4)	< 0.0001
Static compliance^4^ (mL/cmH_2_O)	32.8 (26.3–41.7)	33.6 (26.9–42)	30.7 (24.1–39.9)	0.001
Dynamic compliance^5^ (mL/cmH_2_O)	16.7 (13.6–21)	17 (14.1–21.4)	15.2 (12.3–19.5)	< 0.0001
Blood gas, median (IQR)				
pH	7.4 (7.3–7.4)	7.4 (7.3–7.4)	7.4 (7.3–7.4)	< 0.0001
PaCO_2_ (mmHg)	43 (37–49)	42 (37–48)	45 (40–52)	< 0.0001
PaO_2_/FiO_2_ (mmHg)	145.7 (101.7–200)	152.2 (107–205)	128.3 (87.5–177.5)	< 0.0001
< 150 mmHg, *n* (%)	1106 (51.8%)	796 (48.4%)	310 (63.1%)	< 0.0001
HCO_3_ (mmol/L)	25 (22–27)	24 (22–27)	25 (22–28)	0.001
Lactate (mmol/L)	1.3 (1–1.7)	1.3 (1–1.7)	1.3 (1–1.8)	0.012
Biology, median (IQR)				
Lymphocyte count (× 10^9^/L)	0.8 (0.5–1.1)	0.8 (0.5–1.1)	0.8 (0.6–1.2)	0.294
Thrombocyte count (× 10^9^/L)	225 (167–292.5)	223 (165–291)	227 (170.2–296)	0.367
Total bilirubin (mg/dL)	0.58 (0.41–0.89)	0.58 (0.41–0.89)	0.58 (0.41–0.89)	0.245
Serum creatinine (mg/dL)	0.94 (0.71–1.39)	0.92 (0.7–1.38)	0.98 (0.74–1.46)	0.033
D-dimer (µg/L)	1913 (1100–4219)	1844 (1038.5–4212.2)	2220 (1237–4262)	0.158
CRP (mg/L)	186.4 (121.2–266.5)	180 (119–261.4)	202.4 (136.1–276)	0.021
Procalcitonin (ng/mL)	0.7 (0.3–2.2)	0.6 (0.3–2)	0.9 (0.4–2.9)	0.01
hsTroponine T (ng/L)	23 (12–63.2)	22 (11.3–58.6)	31.4 (14.1–95.2)	0.003

*IQR* interquartile range, *SOFA* sequential organ failure assessment, *SAPS II* simplified acute physiology score II, *PaCO*_*2*_ arterial partial pressure in carbon dioxide, *PaO*_*2*_ arterial partial pressure in oxygen, *FiO*_*2*_ fraction inspired in oxygen, *CRP* C-reactive protein

^1^Defined as plateau pressure—PEEP. If plateau pressure was missing, peak pressure was considered instead

^2^Mechanical power (J/min) = 0.098 × tidal volume × respiratory rate × (peak pressure − 1/2 × driving pressure). If not specified, peak pressure was considered equal to plateau pressure

^3^Defined as (minute ventilation × PaCO2) − (predicted bodyweight × 100 × 37.5)

^4^Normalized for ideal body weight. Defined as tidal volume/(Plateau pressure − PEEP)

^5^Normalized for ideal body weight. Defined as tidal volume/(Peak pressure − PEEP)

### Prone position support

Among the 2137 patients analyzed, 1504 (70.4%) patients were subjected to prone positioning during the ICU stay with a median number of 4 [2–6] PP sessions and a median duration of 20 [16–32] h in the first 48 h.

At Day-1, 491 patients (23%) were placed in PP, constituting the early PP group. The distribution of patients per region is detailed in the Additional file 1: Table S1. Then, 1013 patients (47.4%) were proned after Day-1 with a median delay of 3 [2–5] days after ICU admission, and 633 (29.6%) were never subjected to PP. Those 1646 patients (77%) were classified as the non-early PP group. Characteristics of both groups at Day-1 are summarized in Table 1.

In the early PP group, patients were more obese (54.8% vs. 41.4%, *p* < 0.0001) and had a higher rate of treated hypertension (55.2% vs. 48.3%, *p* = 0.005). Median PaO_2_/FiO_2_ ratio was lower in the early PP group (128.3 [87.5–177.5] mmHg vs. 152.2 [107–205] mmHg, *p* < 0.0001) as well as the respiratory static compliance (30.7 [24.1–39.9] mL/cmH_2_0 vs. 33.6 [26.9–42] mL/cmH_2_0, *p* = 0,001). In the whole cohort, 181 (36.9%) patients of the early PP group had a PaO_2_/FiO_2_ ratio > 150 mmHg when placement in prone position was initiated. On the opposite, 796 (48.4%) patients with PaO_2_/FiO_2_ ratio < 150 mmHg at Day-1 were not placed in PP.

The median number of prone sessions was 3 [2–6] in the non-early PP group, with a median duration of 17 [16–23] h during the first 48 h versus 4 [2–7] number of prone sessions with a duration of 20 [16–32] h in the early PP group (*p* < 0.0001).

### Outcomes

#### In the whole cohort

In unadjusted analysis, mortality at Day-28, Day-60 and Day-90 were 30.5%, 35.4% and 35.9%, respectively, in the complete cohort study. Mortality was significatively lower in the non-early PP group compared to the early PP group as shown in Table 2. More patients needed adjunctive therapies (ECMO, ECCO_2_R, inhaled nitric oxide) in the early PP group. The static compliance, the PaO_2_/FiO_2_ ratio and the SOFA score at Day-3, Day-5 and Day-7 were worse in the early PP group. In the whole cohort, ventilatory parameters did not improve during the first 7 days after ICU admission.

**Table 2:** Primary and secondary outcomes

Outcome	All patients (*n* = 2137)	No early prone position group (*n* = 1646)	Early prone position group (*n* = 491)	*p*
Primary outcome				
Mortality at Day-60, *n* (%)	756 (35.4%)	563 (34.2%)	193 (39.3%)	0.038
Secondary outcomes				
Mortality, *n* (%)				
At Day-28	652 (30.5%)	482 (29.3%)	170 (34.6%)	0.024
At Day-90	767 (35.9%)	574 (34.9%)	193 (39.3%)	0.072
Ventilatory-free days util Day-28, median (IQR)	6 (0–16)	7 (0–17)	0 (0–14)	< 0.001
Extracorporeal membrane oxygenation, *n* (%)	221 (10.4%)	151 (9.2%)	70 (14.3%)	0.001
Extracorporeal CO_2_ removal, *n* (%)	10 (0.7%)	7 (0.7%)	3 (0.9%)	0.719
Inhaled nitric oxide, *n* (%)	412 (19.3%)	286 (17.4%)	126 (25.7%)	< 0.0001
Static compliance (mL/cmH_2_O), median (IQR)				
At Day-3	33.6 (25.7–42.5)	34.4 (26.7–43.6)	31.4 (23.4–40)	< 0.001
At Day-5	31.4 (24.3–40)	32.3 (24.7–40.8)	29.4 (22.6–39.7)	0.011
At Day-7	31 (22.9–40)	31.4 (23.3–40.4)	29.4 (22.1–38.8)	0.105
SOFA score, median (IQR)				
At Day-7	9 (7–12)	9 (7–11)	10 (8–12)	0.002
At Day-21	8 (6.2–11)	8 (7–12)	8 (5–11)	0.638
At Day-28	8 (6.5–11)	8 (6–11)	9 (7–11)	0.905
PaO_2_/FiO_2_ ratio (mmHg), median (IQR)				
At Day-3	158.3 (118.3–213.3)	162.9 (121.2–220)	148 (108.7–192.5)	< 0.0001
At Day-5	155 (113.3–205.5)	158.3 (115–208.6)	140 (106–191.4)	0.001
At Day-7	157.1 (114–205)	158.3 (116–209.3)	150 (104.4–187.5)	0.002
Number of prone sessions during ICU stay, median (IQR)	3 (2–6)	3 (2–6)	4 (2–7)	< 0.0001
Delay between ICU admission and 1st prone position, days, median (IQR)	2 [0–4]	3 [2–5]	0 [0–0]	

*IQR* interquartile range, *SOFA* sequential organ failure assessment, *PaO*_*2*_ arterial partial pressure in oxygen, *FiO*_*2*_ fraction inspired in oxygen, *ICU* intensive care unit

After propensity score adjustment, results were analyzed in both complete case analysis including 944 patients and in multiple imputation analysis with all baseline population of 2137 patients, supplied in the Additional file 1: Table S2. Baseline characteristics before and after weighted-propensity score analysis are provided in the Additional file 1: Table S3.

**Fig. 2:**
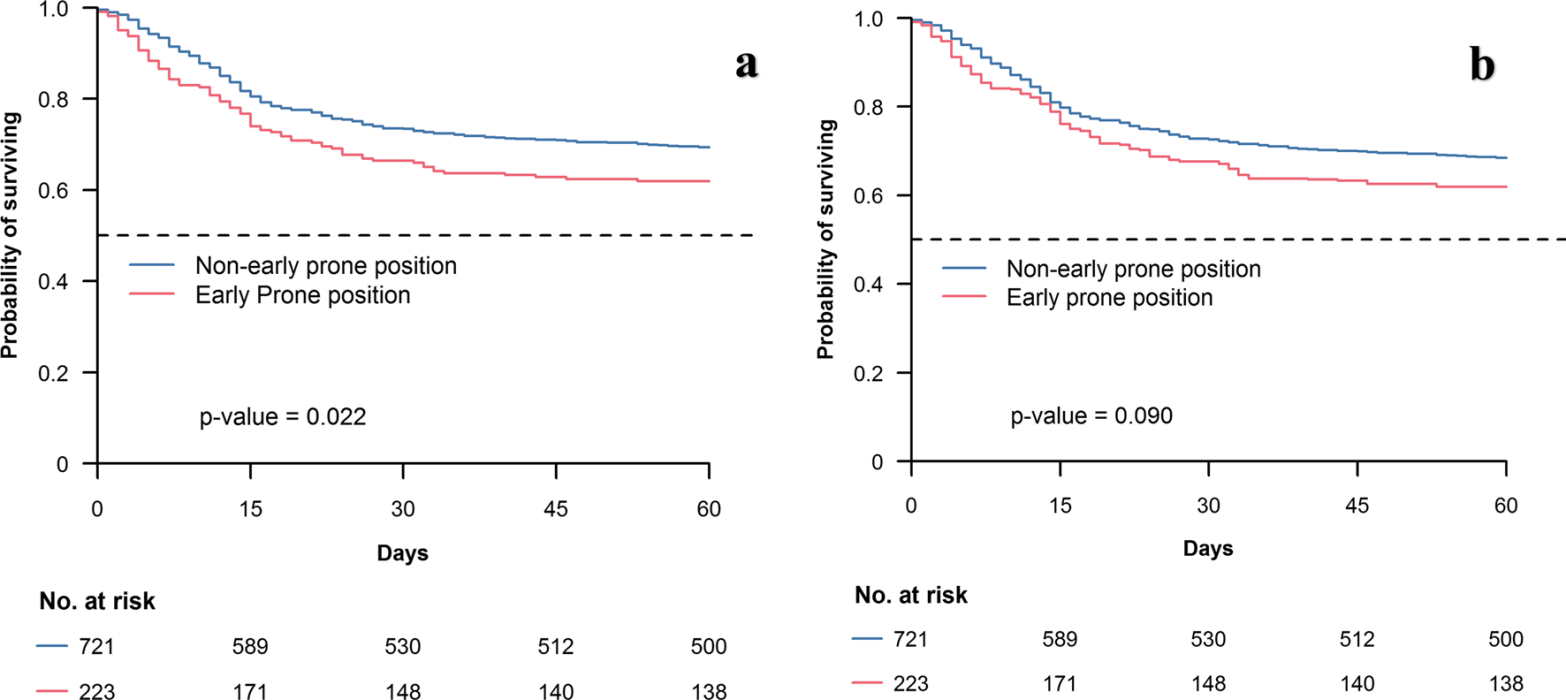
**a** Kaplan–Meier curves according to prone status in ICU at Day-1 before weighting adjustment in complete case population. **b** Kaplan–Meier curves according to prone status in ICU at Day-1 after weighting adjustment in complete case population. *ICU* intensive care unit

**Fig. 3:**
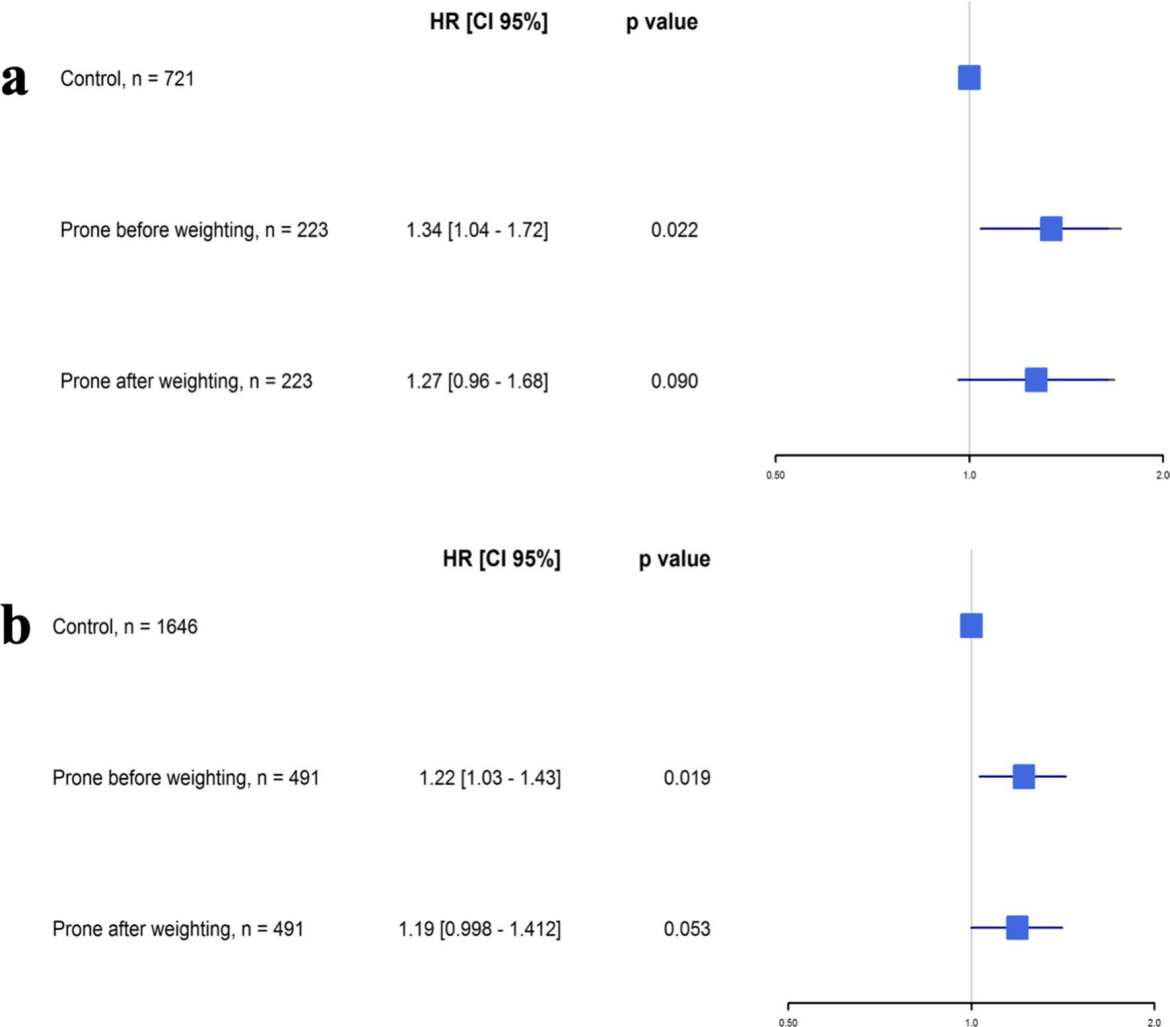
**a** Forest plot: hazard ratio according to prone status in ICU at Day-1 before and after weighting in complete case population. **b** Hazard ratio according to prone status in ICU at Day-1 before and after weighting in baseline population. *ICU* intensive care unit, *HR* hazard ratio

After weighting, no significant difference in Day-60 mortality was found between the two study groups, in both analysis (hazard ratio (HR) 1.34 [0.96–1.68], *p* = 0.09 in complete case analysis and 1.19 [0.998–1.412], *p* = 0.053 in multiple imputation analysis) as illustrated in Figs. 2 and 3. Mortality at Day-28 and Day-90 was also similar between the two study groups after weighted-propensity score analysis.

#### In the subgroups

In the subgroups of ARDS patients according to their PaO_2_/FiO_2_ more or less than 150 at Day-1, mortality was higher in patients with PaO_2_/FiO_2_ less than 150 mmHg (Table 3).

**Table 3:** Subgroups analysis

Variable	PaO_2_/FiO_2_ ratio at Day-1		*p*
	≥ 150 mmHg (*n* = 1031)	< 150 mmHg (*n* = 1106)	
Mortality Day-28, *n* (%)	271 (26.3%)	381 (34.4%)	< 0.0001
Mortality Day-60	312 (30.3%)	444 (40.1%)	< 0.0001
Mortality Day-90	319 (30.9%)	448 (40.5%)	< 0.0001
Invasive ventilation-free days up to Day-28 (days), median (IQR)	9 (0–18)	0 (0–14)	< 0.0001

Time between ICU admission and first prone session			
	After 24 h (*n* = 1013)	Before 24 h (*n* = 491)	
Mortality Day-28, *n* (%)	339 (33.5%)	170 (34.6%)	0.656
Mortality Day-60	403 (39.8%)	193 (39.3%)	0.86
Mortality Day-90	410 (40.5%)	193 (39.3%)	0.665
Invasive ventilation-free days up to Day-28 (days), median (IQR)	0 (0–13)	0 (0–14)	0.415

*IQR* interquartile range, *PaO*_*2*_ arterial partial pressure in oxygen, *FiO*_*2*_ fraction inspired in oxygen, *ICU* Intensive care unit

Among the 1504 patients who received prone positioning during their ICU stay, an early PP was not associated with a reduction of mortality nor an increase in ventilator-free-days up to Day-28, as shown in Table 3. After propensity score adjustment in the subgroup of severely hypoxemic patients (PaO_2_/FiO_2_ ratio less than 150 mmHg) at Day-1, results were analyzed in both complete case analysis including 474 patients and in multiple imputation analysis with all baseline subgroup population of 1106 patients, supplied in the Additional file 1: Table S4. Subgroup baseline characteristics before and after weighted-propensity score analysis are provided in the Additional file 1: Table S5. After weighting, no significant difference in Day-60 mortality was found between the non-early PP and the early PP groups, in both analysis (hazard ratio (HR) 1.12 [0.78–1.59], *p* = 0.55 in complete case analysis and 1.13 [0.9–1.42], *p* = 0.28 in multiple imputation analysis) as illustrated in Additional file 1: Figs. S3 and S4.

However, in the subgroup of non-severely hypoxemic patients (PaO_2_/FiO_2_ ratio more than 150 mmHg) at Day-1, an early PP seemed to be associated to higher Day-60 mortality with a significant difference between the two study groups in both analysis (hazard ratio (HR) 1.7 [1.05–2.77], *p* = 0.03 in complete case analysis and 1.7 [1.16–2.47], *p* = 0.006 in multiple imputation analysis) as illustrated in Additional file 1: Figs. S6 and S7.

## Discussion

In this secondary analysis of a multicenter observational cohort study, our results show that PP was widely used across European ICUs during the COVID-19 pandemic, with 70% of patients intubated at ICU admission placed in prone position during their ICU stay. This rate contrasts with the results of the Lung Safe study and Apronet studies published before this pandemic, reporting less than 15% use of PP in ARDS of all-causes worldwide [8, 21]. Interestingly, our study highlights that prone positioning was not always used according to international guidelines [7, 22]. As a result, a large proportion of patients (37%) was placed in PP despite a PaO_2_/FiO_2_ ratio higher than 150 mmHg. In addition, approximately 50% of patients were not placed in PP at Day-1 despite PaO_2_/FiO_2_ ratio lower than 150 mmHg. Those findings are consistent with results of previous studies [11, 12]. In a recent observational study, Mathews et al. reported that 44% of intubated patients with a PaO_2_/FiO_2_ ratio less than 100 mmHg were not placed in PP during the first 2 days, and only 30% of patients experienced proning during their ICU stay [11]. In a large cohort study of more than 1000 patients, 21% of patients were not placed in PP despite a PaO_2_/FiO_2_ ratio of less than 100 mmHg [13]. Those results highlight the difficulty in this pandemic to properly apply international guidelines. Higher number of ICU beds and higher number of patients per physician or per nurse have previously been associated with a lower use of prone positioning [21]. The intervention of prone positioning in intubated patient requiring experimented staff to do it safely. Work overload, the deterioration of work conditions, the hiring of unexperimented staff and the reorganization of ICU care associated with this pandemic [23, 24] may have contributed to an inadequate use of PP and may explain why patients had not been placed in PP or placed in PP disregarding international guidelines.

Our observational study failed to demonstrate an improvement of survival in intubated patients receiving an early PP at Day-1 compared to non-early PP. Our findings therefore contrast to those reported in another study in mechanically ventilated patients, in which early prone positioning in the first 2 days of ICU admission was associated with a survival benefit in COVID-19-related ARDS [11]. Several reasons may explain these discrepancies. First, definition of treatment group was different between studies. In our study, treatment groups were defined according to their PP status at Day-1 and not according to their PP status in the first 48 h after admission. In order to respect the validity of the propensity score using, our study was designed to analyze a potential survival benefit of prone positioning during the first 24 h of ICU admission. Although the median delay between ICU admission and the first prone positioning in the non-early PP group was 3 days, we could have failed to demonstrate a benefit because approximately 25% of patients in this group had been finally placed in PP during Day-2. Those patients would have been referred as PP group in Mathews et al. study [11]. Consequently, our results suggest no additional outcomes’ improvement supporting very early PP during the first 24 h of ICU admission. Second, our study enrolled all intubated ARDS patients and more than a third of patients placed in PP had a PaO_2_/FiO_2_ ratio higher than 150 mmHg. The Proseva trial showed survival benefit with PP in moderate to severe selected patients with a PaO_2_/FiO_2_ ratio less than 150 mmHg with a PEEP ≥ 10 cmH_2_O and FiO_2_ ≥ 0.6 under standardized mechanical ventilation before inclusion [6]. Even if PP is supposed to limit the extent of lung injuries induced by ventilation in ARDS patients with various degrees of severity, the potential survival benefit in patients with PaO_2_/FiO_2_ ratio higher than 150 mmHg has not been demonstrated and remains unclear mainly due to under-powered previous studies [25]. Third, a large proportion of patients in the early PP group were placed in PP for less than 16 h in contrast to the Proseva trial showing a benefit in patient placed two time in prone position for at least 16 h during the first 2 days [6]. Similar to previous studies [26, 27], the short duration of PP session could also explain the absence of benefit of PP observed in the early PP group. Fourth, as previously described, the PaO_2_/FiO_2_ ratio is influenced by FiO_2_ and the level of PEEP [28]. In this observational study, mechanical ventilation was not standardized before blood gases analyses which was used to define PaO_2_/FiO_2_ ratio, which may have resulted in greater heterogeneity within groups. Finally, 660 patients were proned after 48 h of ICU admission, representing 43.8% of all proned patients in our cohort, and Guerin et al. found a survival benefit when using prone positioning early after endotracheal intubation (within 48 h) [6]. In Mathews et al.’s study a smaller proportion of patients (19.5%) was initiated on proning after 48 h of ICU admission [11], which might have contributed to greater difference in patient’s care between groups and thus impact mortality. However, impact of timing of prone sessions initiation after endotracheal intubation has not been specifically studied yet and is scarcely described in other randomized control trials assessing proning in ARDS [29–31].

Prone position has been shown to improve blood oxygenation by homogenizing the distribution of pulmonary ventilation/perfusion ratios [32–35]; preventing ventilator induced lung injury by homogenizing the strain to lung tissue associated with mechanical ventilation on inflamed alveoli [36–38] and preserving systemic hemodynamics [39], particularly right ventricular function [40]. However, the clear response to the prone position has remained non-defined. Our results show that patients placed in PP at Day-1 did not improve their ventilatory parameters, including the static compliance and oxygenation during their ICU stay at least until Day-7. In a large cohort of intubated COVID-19 patients, Langer et al. found that prone positioning was associated with immediate oxygenation improvement without any increase of respiratory system compliance [13]. The lack of oxygenation improvement in our study could be due to the timing of assessment of oxygenation. Indeed, we recorded blood gases results daily independently of patients proning status at that time and did not study blood gases evolution during and just after proning. This could be in line with results reported by Langer et al. showing a trend toward worsening of oxygenation after re-supination [13]. Our results considering the lack of improvement of static compliance are consistent with those of Langer et al. contrasting data on non-COVID-19-related ARDS which showed a reduction of driving pressure and plateau pressure when placed in prone position, suggesting better static compliance [35]. This difference of effect of PP on respiratory mechanics between COVID-19 and non-COVID-19-related ARDS possibly highlights different pathophysiologies [41]. Those lack of ventilatory parameters improvements could explain why the median duration of invasive mechanical ventilation in ARDS COVID-19 patients is approximately 12–13 days, longer that previously reported in all-causes ARDS patients included in Lung safe study [4, 20]. It might therefore also be possible that the follow-up of 7 days in our study did not allow us to show a potential ventilatory parameters benefits of prone position due to the short time of the follow-up. Moreover, we hypothesize that the main mechanism of the PP benefit in ARDS related to COVID-19 is the redistribution of pulmonary perfusion leading to higher ventilation perfusion ratios, rather than the recruitment, as reported by another study [12]. This pathophysiological rationale could explain why the mechanical property did not improve during the follow-up of our study.

This study has some limitations. First, only patients admitted in the first COVID wave have been enrolled in this research. Second, it is not a randomized controlled study. Although we used a propensity score adjusting on potential confounders, we cannot guarantee in this observational study that: (1) the standardization of mechanical ventilation at all centers was the same as that used in the positive randomized Proseva trial, (2) the PaO_2_/FiO_2_ ratio used by clinicians to initiate PP was calculated after a standardization of setting PEEP and FiO_2_ level, as previously demonstrated as an important factor to define severity of ARDS patients [28, 42]. Third, despite of the propensity score weighting adjustment, it might be possible that patients in the early PP group were more severe at ICU admission and required a prone positioning earlier than patients in the non-early PP group, leading to confusion bias. Moreover, many patients were proned or not disregarded classical criteria for prone position suggesting that many additional factors (clinical, organizational, etc.) may have played a role in the decision to prone or not. However, those undetermined factors cannot be included in the analysis. Fourth, our study design did not allow us to analyze outcomes in patients respecting the PP status in the first 48 h and after stabilization according the Proseva trial protocol, but only depending on the PP status at Day-1. This choice was made to limit the immortal bias that would result from comparing patients who were placed in PP after Day-1 to patients who did not initiate PP at all (patients placed in PP after Day-1 are part of this subgroup because they did not die earlier). Finally, some patients required up to 20 prone sessions leading to potential complications. Unfortunately, those data were not collected in this study.

## Conclusions

Our results suggest that ICUs across European countries have largely adopted prone positioning in ARDS patients due to COVID-19 regardless of their severity. In this observational study, our data failed to show a survival benefit associated with early prone positioning initiated during the first day of ICU admission compared to prone positioning initiation after Day-1 for all COVID-19 patients requiring invasive mechanical ventilation regardless of their severity. Further studies are needed to identify subgroups of patients with COVID-19-related ARDS who might benefit from early prone positioning.

## Supplementary Information

**Additional file 1.** Additional information about the baseline characteristics and the statistical analysis (file format in .docx). **Additional Tables and Figures.** **Table S1.** Distribution of patients per region included in this study according to their prone position status at Day-1. **Table S2.** Descriptive analysis of baseline population included in propensity score analysis and complete case population. **Table S3.** Descriptive analysis of baseline characteristics before and after weighted-propensity score analysis. **Fig. S1.** Adjustment quality before and after propensity score analysis. **Table S4**. Descriptive subgroup analysis of baseline population with P_a_O_2_/F_i_O_2_ ratio < 150 mmHg at Day-1 included in propensity score analysis and complete case population. **Table S5**. Descriptive subgroup analysis of baseline population characteristics with P_a_O_2_/F_i_O_2_ ratio < 150 mmHg at Day-1 before and after weighted-propensity score analysis. **Fig. S2.** Adjustment quality before and after propensity score analysis in the subgroup of patients with P_a_O_2_/F_i_O_2_ ratio < 150 mmHg at Day-1. **Fig. S3**. **a **Kaplan–Meier curves according to prone status in ICU at Day-1 before weighting adjustment in complete case subgroup population with P_a_O_2_/F_i_O_2_ ratio < 150 mmHg. **b** Kaplan–Meier curves according to prone status in ICU at Day-1 after weighting adjustment in complete case subgroup population with P_a_O_2_/F_i_O_2_ < 150 mmHg. **Fig. S4.** **a **Forest plot: Hazard Ratio according to prone status in ICU at Day-1 before and after weighting in complete case subgroup population with P_a_O_2_/F_i_O_2_ ratio < 150 mmHg. **b** Hazard Ratio according to prone status in ICU at Day-1 before and after weighting in baseline subgroup population with P_a_O_2_/F_i_O_2_ ratio < 150 mmHg. **Table S6**. Descriptive subgroup analysis of baseline population with P_a_O_2_/F_i_O_2_ ratio > 150 mmHg included in propensity score analysis and complete case population. **Table S7.** Descriptive subgroup analysis of baseline population characteristics with P_a_O_2_/F_i_O_2_ ratio > 150 mmHg at Day-1 before and after weighted-propensity score analysis. **Fig. S5.** Adjustment quality before and after propensity score analysis in the subgroup of patients with P_a_O_2_/F_i_O_2_ ratio > 150 mmHg at Day-1. **Fig. S6.** **a** Kaplan–Meier curves according to prone status in ICU at Day-1 before weighting adjustment in complete case subgroup population with P_a_O_2_/F_i_O_2_ ratio > 150 mmHg. **b** Kaplan–Meier curves according to prone status in ICU at Day-1 after weighting adjustment in complete case subgroup population with P_a_O_2_/F_i_O_2_ ratio > 150 mmHg. **Fig. S7.** **a** Forest plot: Hazard Ratio according to prone status in ICU at Day-1 before and after weighting in complete case subgroup population with P_a_O_2_/F_i_O_2_ ratio > 150 mmHg. **b** Hazard Ratio according to prone status in ICU at Day-1 before and after weighting in baseline subgroup population with P_a_O_2_/F_i_O_2_ ratio > 150 mmHg.

## Data Availability

The datasets used and/or analyzed during the current study are available from the corresponding author on reasonable request.

## Abbreviations

COVID-19: Coronavirus disease 2019
PP: Prone position
ICU: Intensive care unit
ARDS: Acute respiratory distress syndrome
RT-PCR: Real-time reverse transcriptase-polymerase chain reaction
BMI: Body mass index
SOFA: Sequential Organ Failure Assessment
PaO_2_: Arterial partial pressure of oxygen
FiO_2_: Fraction inspired of oxygen
SAPS II: Simplified Acute Physiology Score II
PEEP: Positive end-expiratory pressure
ECMO: Extracorporeal membrane oxygenation
ECCO_2_R: Extracorporeal CO_2_ removal
IPTW: Inverse probability of treatment weighting
PS: Propensity score

## References

[1] World Health Organization. Coronarovirus (COVID-19) dashboard. Available at https://covid19.who.int. Accessed 12 May 2022.

[2] Grasselli G, Greco M, Zanella A (2020). Risk factors associated with mortality among patients with COVID-19 in intensive care units in Lombardy, Italy. JAMA Intern Med.

[3] Bhatraju PK, Ghassemieh BJ, Nichols M (2020). Covid-19 in critically ill patients in the Seattle Region—case series. N Engl J Med.

[4] COVID-ICU Group on behalf of the REVA network and the COVID-ICU investigators (2021). Clinical characteristics and day-90 outcomes of 4244 critically ill adults with COVID-19: a prospective cohort study. Intensive Care Med.

[5] Grasselli G, Zangrillo A, Zanella A, Antonelli M, Cabrini L, Castelli A (2020). Baseline characteristics and outcomes of 1591 patients infected with SARS-CoV-2 admitted to ICUs of the Lombardy Region, Italy. JAMA.

[6] Guérin C, Reignier J, Richard JC (2013). Prone positioning in severe acute respiratory distress syndrome. N Engl J Med.

[7] Papazian L, Aubron C, Brochard L (2019). Formal guidelines: management of acute respiratory distress syndrome. Ann Intensive Care.

[8] Guérin C, Beuret P, Constantin JM (2018). A prospective international observational prevalence study on prone positioning of ARDS patients: the APRONET (ARDS Prone Position Network) study. Intensive Care Med.

[9] Alhazzani W, Møller MH, Arabi YM (2020). Surviving Sepsis Campaign: guidelines on the management of critically ill adults with Coronavirus Disease 2019 (COVID-19). Intensive Care Med.

[10] Ferrando C, Suarez-Sipmann F, Mellado-Artigas R (2020). Clinical features, ventilatory management, and outcome of ARDS caused by COVID-19 are similar to other causes of ARDS. Intensive Care Med.

[11] Mathews KS, Soh H, Shaefi S, et al. Prone positioning and survival in mechanically ventilated patients with coronavirus disease 2019-related respiratory failure. Crit Care Med. 2021. 10.1097/CCM.0000000000004938 PMC8277560 33595960

[12] Camporota L, Sanderson B, Chiumello D, et al. Prone position in coronavirus disease 2019 and noncoronavirus disease 2019 acute respiratory distress syndrome: an international multicenter observational comparative study. Crit Care Med. 2021. 10.1097/CCM.0000000000005354 PMC8923275 34582426

[13] Langer T, Brioni M, Guzzardella A (2021). Prone position in intubated, mechanically ventilated patients with COVID-19: a multi-centric study of more than 1000 patients. Crit Care.

[14] Scaramuzzo G, Gamberini L, Tonetti T (2021). Sustained oxygenation improvement after first prone positioning is associated with liberation from mechanical ventilation and mortality in critically ill COVID-19 patients: a cohort study. Ann Intensive Care.

[15] Ranieri VM, Rubenfeld GD, ARDS Definition Task Force (2012). Acute respiratory distress syndrome: the Berlin definition. JAMA.

[16] Yehya N, Harhay MO, Curley MAQ, Schoenfeld DA, Reeder RW (2019). Reappraisal of ventilator-free days in critical care research. Am J Respir Crit Care Med.

[17] Austin PC (2011). An introduction to propensity score methods for reducing the effects of confounding in observational studies. Multivar Behav Res.

[18] Austin PC (2009). Balance diagnostics for comparing the distribution of baseline covariates between treatment groups in propensity-score matched samples. Stat Med.

[19] Vesin A, Azoulay E, Ruckly S (2013). Reporting and handling missing values in clinical studies in intensive care units. Intensive Care Med.

[20] Grambsch PM, Therneau TM (1994). Proportional hazards tests and diagnostics based on weighted residuals. Biometrika.

[21] Bellani G, Laffey JG, Pham T (2016). Epidemiology, patterns of care, and mortality for patients with acute respiratory distress syndrome in intensive care units in 50 countries. JAMA.

[22] Fan E, Del Sorbo L, Goligher EC (2017). An Official American Thoracic Society/European Society of Intensive Care Medicine/Society of Critical Care Medicine Clinical Practice Guideline: mechanical ventilation in adult patients with acute respiratory distress syndrome. Am J Respir Crit Care Med.

[23] Primmaz S, Le Terrier C, Suh N (2020). Preparedness and reorganization of care for coronavirus disease 2019 patients in a swiss ICU: characteristics and outcomes of 129 patients. Crit Care Explor.

[24] Doussot A, Ciceron F, Cerutti E (2020). Prone positioning for severe acute respiratory distress syndrome in COVID-19 patients by a dedicated team: a safe and pragmatic reallocation of medical and surgical work force in response to the outbreak. Ann Surg.

[25] Albert RK (2020). Prone ventilation for patients with mild or moderate acute respiratory distress syndrome. Ann Am Thorac Soc.

[26] Papazian L, Paladini MH, Bregeon F (2001). Is a short trial of prone positioning sufficient to predict the improvement in oxygenation in patients with acute respiratory distress syndrome?. Intensive Care Med.

[27] Guerin C, Gaillard S, Lemasson S (2004). Effects of systematic prone positioning in hypoxemic acute respiratory failure: a randomized controlled trial. JAMA.

[28] Palanidurai S, Phua J, Chan YH, Mukhopadhyay A (2021). Is it time to revisit the PaO_2_/FiO_2_ ratio to define the severity of oxygenation in ARDS ?. Ann Intensive Care.

[29] Gattinoni L, Tognoni G, Pesenti A (2001). Effect of prone positioning on the survival of patients with acute respiratory failure. N Engl J Med.

[30] Mancebo J, Fernández R, Blanch L (2006). A multicenter trial of prolonged prone ventilation in severe acute respiratory distress syndrome. Am J Respir Crit Care Med.

[31] Taccone P, Pesenti A, Latini R (2009). Prone positioning in patients with moderate and severe acute respiratory distress syndrome: a randomized controlled trial. JAMA.

[32] Galiatsou E, Kostanti E, Svarna E (2006). Prone position augments recruitment and prevents alveolar overinflation in acute lung injury. Am J Respir Crit Care Med.

[33] Henderson AC, Sá RC, Theilmann RJ, Buxton RB, Prisk GK, Hopkins SR (2013). The gravitational distribution of ventilation-perfusion ratio is more uniform in prone than supine posture in the normal human lung. J Appl Physiol.

[34] Lamm WJ, Graham MM, Albert RK (1994). Mechanism by which the prone position improves oxygenation in acute lung injury. Am J Respir Crit Care Med.

[35] Guérin C, Albert RK, Beitler J (2020). Prone position in ARDS patients: why, when, how and for whom. Intensive Care Med.

[36] Pelosi P, Tubiolo D, Mascheroni D (1998). Effects of the prone position on respiratory mechanics and gas exchange during acute lung injury. Am J Respir Crit Care Med.

[37] Gattinoni L, Taccone P, Carlesso E, Marini JJ (2013). Prone position in acute respiratory distress syndrome. Rationale, indications, and limits. Am J Respir Crit Care Med.

[38] Guerin C, Baboi L, Richard JC (2014). Mechanisms of the effects of prone positioning in acute respiratory distress syndrome. Intensive Care Med.

[39] Jozwiak M, Teboul JL, Anguel N (2013). Beneficial hemodynamic effects of prone positioning in patients with acute respiratory distress syndrome. Am J Respir Crit Care Med.

[40] Vieillard-Baron A, Charron C, Caille V, Belliard G, Page B, Jardin F (2007). Prone positioning unloads the right ventricle in severe ARDS. Chest.

[41] Chiumello D, Busana M, Coppola S (2020). Physiological and quantitative CT-scan characterization of COVID-19 and typical ARDS: a matched cohort study. Intensive Care Med.

[42] Aboab J, Louis B, Jonson B, Brochard L (2006). Relation between PaO_2_/FIO_2_ ratio and FIO_2_: a mathematical description. Intensive Care Med.

