# Supplementary material for: Early prone positioning in acute respiratory distress syndrome related to COVID-19: a propensity score analysis from the multicentric cohort COVID-ICU network—the ProneCOVID study
Source: Crit Care. 2022 Mar 24;26:71. doi: 10.1186/s13054-022-03949-7 (PMC8944409; doi:10.1186/s13054-022-03949-7)

**ADDITIONAL FILE 1**

**Early Prone Positioning in acute respiratory distress syndrome related to COVID-19: a propensity score analysis from the multicentric cohort COVID-ICU network. The ProneCOVID study.**

Christophe Le Terrier, MD^1^; Florian Sigaud, MD^2^; Said Lebbah, MD^3^; Luc Desmedt, MD^4^; David Hajage, MD^3^; Claude Guérin, MD^5^; Jérôme Pugin, MD^1^; Steve Primmaz, MD^1^; Nicolas Terzi, MD^2^; COVID-ICU Group on behalf of the REVA Network and the COVID-ICU Investigators*

* COVID-ICU Investigators are listed at the end of the manuscript.

**^1^** Division of Intensive care, Geneva University Hospitals and the University of Geneva Faculty of Medicine, Geneva, Switzerland

**^2^** Division of Intensive Care, Grenoble Alpes University Hospital, Grenoble, France

**^3^** AP-HP, Département de Santé Publique, Centre de Pharmaco-épidémiologie, Paris, France

**^4^** Medical Intensive Care unit, Nantes Hôtel-Dieu University Hospital, Nantes, France

**^5^** Division of Intensive Care, Edouard Herriot Hospital, Lyon, France

**Word count**: Abstract 315; Manuscript 3922; 3 Tables; 3 Figures; 1 Additional file

**Running title**: *Prone positioning in ARDS due to COVID-19.*

**Submitted to:** *Critical Care*

**Keywords:** Acute respiratory distress syndrome – Intubation – COVID-19 – Mortality – Prone position– Intensive care unit.

**Corresponding author**

Nicolas Terzi, MD - PhD

Medical Intensive Care Unit

Grenoble Alpes University Hospital

Avenue Maquis du Grésivaudan

38700 La Tronche, France

Tel.: +33 476 76 71 09

E-mail [nterzi@chu-grenoble.fr](mailto:nterzi@chu-grenoble.fr)

**COVID-ICU collaborative group**

Investigators are listed at the end of the manuscript

E-mail: covid.icu@gmail.com

**Contents**

**Additional Tables and Figures**

**Table 1** Distribution of patients per region included in this study according to their prone position status at Day-1 4

**Table 2** Descriptive analysis of baseline population included in propensity score analysis and complete case population 5

**Table 3** Descriptive analysis of baseline characteristics before and after weighted-propensity score analysis

7

**Figure 1** Adjustment quality before and after propensity score analysis. 9

**Table 4**. Descriptive subgroup analysis of baseline population with P_a_O_2_/F_i_O_2_ ratio <150 mmHg at Day-1 included in propensity score analysis and complete case population. 10

**Table 5**. Descriptive subgroup analysis of baseline population characteristics with P_a_O_2_/F_i_O_2_ ratio <150 mmHg at Day-1 before and after weighted-propensity score analysis 12

**Figure 2.** Adjustment quality before and after propensity score analysis in the subgroup of patients with P_a_O_2_/F_i_O_2_ ratio < 150 mmHg at Day-1 14

**Figure 3. a.** Kaplan Meier curves according to prone status in ICU at Day-1 before weighting adjustment in complete case subgroup population with P_a_O_2_/F_i_O_2_ ratio <150 mmHg. **b.** Kaplan Meier curves according to prone status in ICU at Day-1 after weighting adjustment in complete case subgroup population with P_a_O_2_/F_i_O_2_ <150 mmHg. 15

**Figure 4. a.** Forest plot: Hazard Ratio according to prone status in ICU at Day-1 before and after weighting in complete case subgroup population with P_a_O_2_/F_i_O_2_ ratio <150 mmHg. **b.** Hazard Ratio according to prone status in ICU at Day-1 before and after weighting in baseline subgroup population with P_a_O_2_/F_i_O_2_ ratio <150 mmHg. 16

**Table 6**. Descriptive subgroup analysis of baseline population with P_a_O_2_/F_i_O_2_ ratio >150 mmHg included in propensity score analysis and complete case population. 17

**Table 7**. Descriptive subgroup analysis of baseline population characteristics with P_a_O_2_/F_i_O_2_ ratio >150 mmHg at Day-1 before and after weighted-propensity score analysis. 19

**Figure 5.** Adjustment quality before and after propensity score analysis in the subgroup of patients with P_a_O_2_/F_i_O_2_ ratio >150 mmHg at Day-1. 21

**Figure 6. a.** Kaplan Meier curves according to prone status in ICU at Day-1 before weighting adjustment in complete case subgroup population with P_a_O_2_/F_i_O_2_ ratio >150 mmHg. **b.** Kaplan Meier curves according to prone status in ICU at Day-1 after weighting adjustment in complete case subgroup population with P_a_O_2_/F_i_O_2_ ratio >150 mmHg. 22

**Figure 7. a.** Forest plot: Hazard Ratio according to prone status in ICU at Day-1 before and after weighting in complete case subgroup population with P_a_O_2_/F_i_O_2_ ratio >150 mmHg. **b.** Hazard Ratio according to prone status in ICU at Day-1 before and after weighting in baseline subgroup population with P_a_O_2_/F_i_O_2_ ratio >150 mmHg. 23

**Table 1.** Distribution of patients per region included in this study according to their prone position status at Day-1

| **Region** | **Non-early prone position group** | **Early prone position group** | **Total** |
| --- | --- | --- | --- |
| Ile-de-France, France | 935 (56.8%) | 267 (54.4%) | 1202 (56.2%) |
| Grand Est, France | 112 (6.8%) | 35 (7.1%) | 147 (6.9%) |
| Hauts-de-France, France | 86 (5.2%) | 14 (2.9%) | 100 (4.7%) |
| Auvergne-Rhône-Alpes, France | 67 (4.1%) | 32 (6.5%) | 99 (4.6%) |
| Genève, Switzerland | 73 (4.4%) | 18 (3.7%) | 91 (4.3%) |
| Pays de la Loire, France | 69 (4.2%) | 21 (4.3%) | 90 (4.2%) |
| Normandie, France | 47 (2.9%) | 11 (2.2%) | 58 (2.7%) |
| Nouvelle-Aquitaine, France | 51 (3.1%) | 7 (1.4%) | 58 (2.7%) |
| Centre-Val de Loire, France | 44 (2.7%) | 13 (2.6%) | 57 (2.7%) |
| Provence-Alpes-Côte d'Azur, France | 43 (2.6%) | 14 (2.9%) | 57 (2.7%) |
| Liège, Belgian | 29 (1.8%) | 22 (4.5%) | 51 (2.4%) |
| Bretagne, France | 28 (1.7%) | 11 (2.2%) | 39 (1.8%) |
| Bourgogne-Franche-Comté, France | 17 (1%) | 9 (1.8%) | 26 (1.2%) |
| Occitanie, France | 22 (1.3%) | 4 (0.8%) | 26 (1.2%) |
| Guadeloupe, France | 8 (0.5%) | 4 (0.8%) | 12 (0.6%) |
| Martinique, France | 9 (0.5%) | 2 (0.4%) | 11 (0.5%) |
| Charleroi, Belgian | 4 (0.2%) | 6 (1.2%) | 10 (0.5%) |
| La Réunion, France | 2 (0.1%) | 0 (0%) | 2 (0.1%) |
| Polynésie française, France | 0 (0%) | 1 (0.2%) | 1 (0%) |
| Total | 1646 (77%) | 491 (23%) | 2137 (100%) |

**Table 2**. Descriptive analysis of baseline population included in propensity score analysis and complete case population

|  |  | **Baseline population (n = 2137)** | |  | **Complete case population (n = 944)** | |  |
| --- | --- | --- | --- | --- | --- | --- | --- |
| **label** | **N (NA)** | **Early PP (n = 491)** | **Non-early PP (n = 1646)** | **p** | **Early PP (n = 223)** | **Non-early PP (n = 721)** | **p** |
| Age, years, <40 | 2137 (0) | 63 (4%) | 25 (5%) | 0.5892 | 31 (4%) | 14 (6%) | 0.6586 |
| Age, years, 40 - 59 | 2137 (0) | 579 (35%) | 176 (36%) | 0.5892 | 263 (36%) | 79 (35%) | 0.6586 |
| Age, years, 60 - 74 | 2137 (0) | 806 (49%) | 236 (48%) | 0.5892 | 354 (49%) | 106 (48%) | 0.6586 |
| Age, years, ≥ 75 | 2137 (0) | 198 (12%) | 54 (11%) | 0.5892 | 73 (10%) | 24 (11%) | 0.6586 |
| Genre, Male | 2127 (10) | 1242 (76%) | 356 (73%) | 0.2382 | 562 (78%) | 164 (74%) | 0.1726 |
| Genre, Female | 2127 (10) | 398 (24%) | 131 (27%) | 0.2382 | 159 (22%) | 59 (26%) | 0.1726 |
| Frailty clinical scale, 1 - 3 | 1935 (202) | 1286 (87%) | 398 (88%) | 0.4226 | 630 (87%) | 198 (89%) | 0.3133 |
| Frailty clinical scale, 4 | 1935 (202) | 133 (9%) | 38 (8%) | 0.4226 | 63 (9%) | 21 (9%) | 0.3133 |
| Frailty clinical scale, 5 - 9 | 1935 (202) | 66 (4%) | 14 (3%) | 0.4226 | 28 (4%) | 4 (2%) | 0.3133 |
| SOFA Cardiovascular system, >= 3 | 2108 (29) | 1007 (62%) | 305 (63%) | 0.5467 | 486 (67%) | 145 (65%) | 0.5087 |
| SOFA Coagulation, >= 3 | 2072 (65) | 14 (1%) | 3 (1%) | 0.7769 | 5 (1%) | 1 (0%) | 1.0000 |
| SOFA Renal, >= 3 | 2080 (57) | 137 (9%) | 39 (8%) | 0.8723 | 62 (9%) | 19 (9%) | 0.9706 |
| Immunodepression | 2117 (20) | 120 (7%) | 34 (7%) | 0.7876 | 57 (8%) | 11 (5%) | 0.1334 |
| Treated hypertension | 2113 (24) | 786 (48%) | 269 (55%) | 0.0076 | 338 (47%) | 125 (56%) | 0.0166 |
| Diabetes | 2115 (22) | 446 (27%) | 155 (32%) | 0.0528 | 202 (28%) | 66 (30%) | 0.6475 |
| Body mass index, kg/m2, < 30 | 1998 (139) | 902 (59%) | 208 (45%) | <0.0001 | 425 (59%) | 105 (47%) | 0.0050 |
| Body mass index, kg/m2, 30 - 39 | 1998 (139) | 569 (37%) | 213 (46%) | <0.0001 | 267 (37%) | 103 (46%) | 0.0050 |
| Body mass index, kg/m2, ≥ 40 | 1998 (139) | 67 (4%) | 39 (8%) | <0.0001 | 29 (4%) | 15 (7%) | 0.0050 |
| PaO_2_/FiO_2_ ratio, 201 - 300 mmHg | 2137 (0) | 437 (27%) | 83 (17%) | <0.0001 | 211 (29%) | 39 (17%) | <0.0001 |
| PaO_2_/FiO_2_ ratio, 101 - 200 mmHg | 2137 (0) | 857 (52%) | 241 (49%) | <0.0001 | 367 (51%) | 105 (47%) | <0.0001 |
| PaO_2_/FiO_2_ ratio, ≤ 100 mmHg | 2137 (0) | 352 (21%) | 167 (34%) | <0.0001 | 143 (20%) | 79 (35%) | <0.0001 |
| Days between first signs and ICU admission, < 4 | 2005 (132) | 200 (13%) | 63 (14%) | 0.9401 | 101 (14%) | 27 (12%) | 0.6618 |
| Days between first signs and ICU admission, 4 - 7 | 2005 (132) | 541 (35%) | 165 (35%) | 0.9401 | 254 (35%) | 76 (34%) | 0.6618 |
| Days between first signs and ICU admission, ≥ 8 | 2005 (132) | 798 (52%) | 238 (51%) | 0.9401 | 366 (51%) | 120 (54%) | 0.6618 |
| Lymphocyte count, × 10^9/L | 1817 (320) | 1 (2) | 2 (6) | 0.2940 | 1 (1) | 2 (8) | 0.2470 |
| SAPS II score | 1980 (157) | 45 (17) | 45 (16) | 0.7017 | 45 (17) | 46 (17) | 0.6813 |
| Static compliance, day 1, mL/cmH2O, < 30 | 1499 (638) | 390 (34%) | 168 (46%) | 0.0006 | 245 (34%) | 103 (46%) | 0.0029 |
| Static compliance, day 1, mL/cmH2O, 30 - 39 | 1499 (638) | 387 (34%) | 108 (29%) | 0.0006 | 243 (34%) | 67 (30%) | 0.0029 |
| Static compliance, day 1, mL/cmH2O, ≥ 40 | 1499 (638) | 354 (31%) | 92 (25%) | 0.0006 | 233 (32%) | 53 (24%) | 0.0029 |
| Bacterial coinfection at ICU admission | 2077 (60) | 93 (6%) | 37 (8%) | 0.1427 | 40 (6%) | 18 (8%) | 0.1702 |
| ICU admission period, before March 28 | 2137 (0) | 936 (57%) | 256 (52%) | 0.0642 | 414 (57%) | 126 (57%) | 0.8087 |
| ICU admission period, after March 29 | 2137 (0) | 710 (43%) | 235 (48%) | 0.0642 | 307 (43%) | 97 (43%) | 0.8087 |

SAPS: Simplified Acute Physiology Score; SOFA: Sequential Organ Failure Assessment.

Data are mean (SD) for continuous variables and frequency (percentage) for categorical variables. N (NA): Number of observations (number of missing data).

p = p value of comparison tests: Wilcoxon sum rank test for continuous variables and Chi square test or Fisher’s exact test, as appropriate, for categorical variables.

**Table 3***.* Descriptive analysis of baseline characteristics before and after weighted-propensity score analysis

|  | **Complete case population (n = 944)** | | | | | | **Baseline population, multiple imputation (n = 2137)** | | | | | |
| --- | --- | --- | --- | --- | --- | --- | --- | --- | --- | --- | --- | --- |
|  | **Before weighting** | | | **After weighting** | | | **Before weighting** | | | **After weighting** | | |
| **label** | **Non-early PP (n = 721)** | **Early PP (n = 223)** | **SMD** | **Non-early PP (n = 721)** | **Early PP (n = 223)** | **SMD** | **Non-early PP (n = 1646)** | **Early PP (n = 491)** | **SMD** | **Non-early PP (n = 1646)** | **Early PP (n = 491)** | **SMD** |
| Age, years, <40 | 4% | 6% | 0.088 | 5% | 4% | 0.035 | 4% | 5% | 0.061 | 4% | 4% | 0.022 |
| Age, years, 40 - 59 | 36% | 35% | 0.022 | 36% | 36% | 0.002 | 32% | 34% | 0.027 | 33% | 33% | 0.005 |
| Age, years, 60 - 74 | 49% | 48% | 0.031 | 49% | 50% | 0.027 | 49% | 48% | 0.018 | 49% | 50% | 0.020 |
| Age, years, ≥ 75 | 10% | 11% | 0.021 | 10% | 10% | 0.018 | 15% | 13% | 0.047 | 14% | 14% | 0.021 |
| Genre, Male | 78% | 74% | 0.103 | 77% | 76% | 0.027 | 76% | 73% | 0.060 | 75% | 76% | 0.020 |
| Genre, Female | 22% | 26% | 0.103 | 23% | 24% | 0.027 | 24% | 27% | 0.060 | 25% | 24% | 0.020 |
| Frailty clinical scale, 1 - 3 | 87% | 89% | 0.043 | 88% | 87% | 0.009 | 86% | 88% | 0.057 | 87% | 87% | 0.019 |
| Frailty clinical scale, 4 | 9% | 9% | 0.024 | 9% | 8% | 0.029 | 9% | 8% | 0.024 | 9% | 9% | 0.007 |
| Frailty clinical scale, 5 - 9 | 4% | 2% | 0.126 | 3% | 5% | 0.057 | 5% | 3% | 0.066 | 4% | 4% | 0.025 |
| SOFA Cardiovascular system, >= 3 | 67% | 65% | 0.050 | 67% | 68% | 0.026 | 62% | 64% | 0.034 | 62% | 63% | 0.016 |
| SOFA Coagulation, >= 3 | 1% | 0% | 0.032 | 1% | 1% | 0.051 | 1% | 1% | 0.023 | 1% | 1% | 0.053 |
| SOFA Renal, >= 3 | 9% | 9% | 0.003 | 9% | 9% | 0.014 | 9% | 8% | 0.010 | 8% | 9% | 0.013 |
| Immunodepression | 8% | 5% | 0.121 | 7% | 7% | 0.016 | 7% | 7% | 0.015 | 7% | 7% | 0.004 |
| Treated hypertension | 47% | 56% | 0.184 | 49% | 51% | 0.030 | 49% | 55% | 0.134 | 50% | 52% | 0.041 |
| Diabetes | 28% | 30% | 0.035 | 28% | 28% | 0.001 | 27% | 32% | 0.101 | 28% | 29% | 0.009 |
| Body mass index, kg/m2, < 30 | 59% | 47% | 0.239 | 56% | 58% | 0.022 | 59% | 46% | 0.263 | 56% | 56% | 0.010 |
| Body mass index, kg/m2, 30 - 39 | 37% | 46% | 0.186 | 39% | 37% | 0.030 | 37% | 46% | 0.185 | 39% | 38% | 0.012 |
| Body mass index, kg/m2, ≥ 40 | 4% | 7% | 0.120 | 5% | 5% | 0.016 | 4% | 8% | 0.161 | 5% | 5% | 0.004 |
| PaO_2_/FiO_2_ ratio, 201 – 300 mmHg | 29% | 17% | 0.281 | 27% | 26% | 0.018 | 27% | 17% | 0.235 | 24% | 23% | 0.024 |
| PaO_2_/FiO_2_ ratio, 101 - 200 mmHg | 51% | 47% | 0.076 | 50% | 52% | 0.034 | 52% | 49% | 0.060 | 51% | 52% | 0.016 |
| PaO_2_/FiO_2_ ratio, ≤ 100 mmHg | 20% | 35% | 0.354 | 23% | 23% | 0.021 | 21% | 34% | 0.285 | 24% | 25% | 0.004 |
| Days between first signs and ICU admission, < 4 | 14% | 12% | 0.056 | 14% | 15% | 0.027 | 9% | 8% | 0.041 | 9% | 9% | 0.007 |
| Days between first signs and ICU admission, 4 - 7 | 35% | 34% | 0.024 | 35% | 38% | 0.060 | 30% | 33% | 0.068 | 30% | 30% | 0.004 |
| Days between first signs and ICU admission, ≥ 8 | 51% | 54% | 0.061 | 51% | 47% | 0.077 | 61% | 59% | 0.041 | 61% | 61% | 0.006 |
| Lymphocyte count, × 10^9/L | 1.0 (1.2) | 2.2 (7.9) | 0.20 | 1.1 (1.3) | 1.2 (4.0) | 0.044 | 1.2 (2.2) | 1.9 (6.6) | 0.145 | 1.3 (2.8) | 1.3 (3.8) | 0.009 |
| SAPS II score | 45 (17) | 46 (17) | 0.011 | 46 (17) | 45 (16) | 0.018 | 45 (17) | 45 (16) | 0.007 | 45 (17) | 46 (17) | 0.022 |
| Static compliance, day 1, mL/cmH2O, < 30 | 34% | 46% | 0.251 | 37% | 35% | 0.031 | 35% | 45% | 0.206 | 37% | 37% | 0.009 |
| Static compliance, day 1, mL/cmH2O, 30 - 39 | 34% | 30% | 0.078 | 33% | 31% | 0.035 | 33% | 30% | 0.076 | 33% | 32% | 0.013 |
| Static compliance, day 1, mL/cmH2O, ≥ 40 | 32% | 24% | 0.191 | 30% | 33% | 0.067 | 32% | 25% | 0.144 | 30% | 31% | 0.022 |
| Bacterial coinfection at ICU admission | 6% | 8% | 0.100 | 6% | 6% | 0.025 | 6% | 8% | 0.072 | 6% | 6% | 0.008 |
| ICU admission period, before March 28 | 57% | 57% | 0.019 | 57% | 58% | 0.004 | 57% | 52% | 0.095 | 56% | 56% | 0.005 |
| ICU admission period, after March 29 | 43% | 43% | 0.019 | 43% | 42% | 0.004 | 43% | 48% | 0.095 | 44% | 44% | 0.005 |

SAPS: Simplified Acute Physiology Score; SOFA: Sequential Organ Failure Assessment.

Data are mean (SD) for continuous variables and percentages for categorical variables; SMD: Standardized Mean difference; After weighting: mean, SD and percentages are weighted by IPTW (Inverse of Probability of Treatment Weighted).


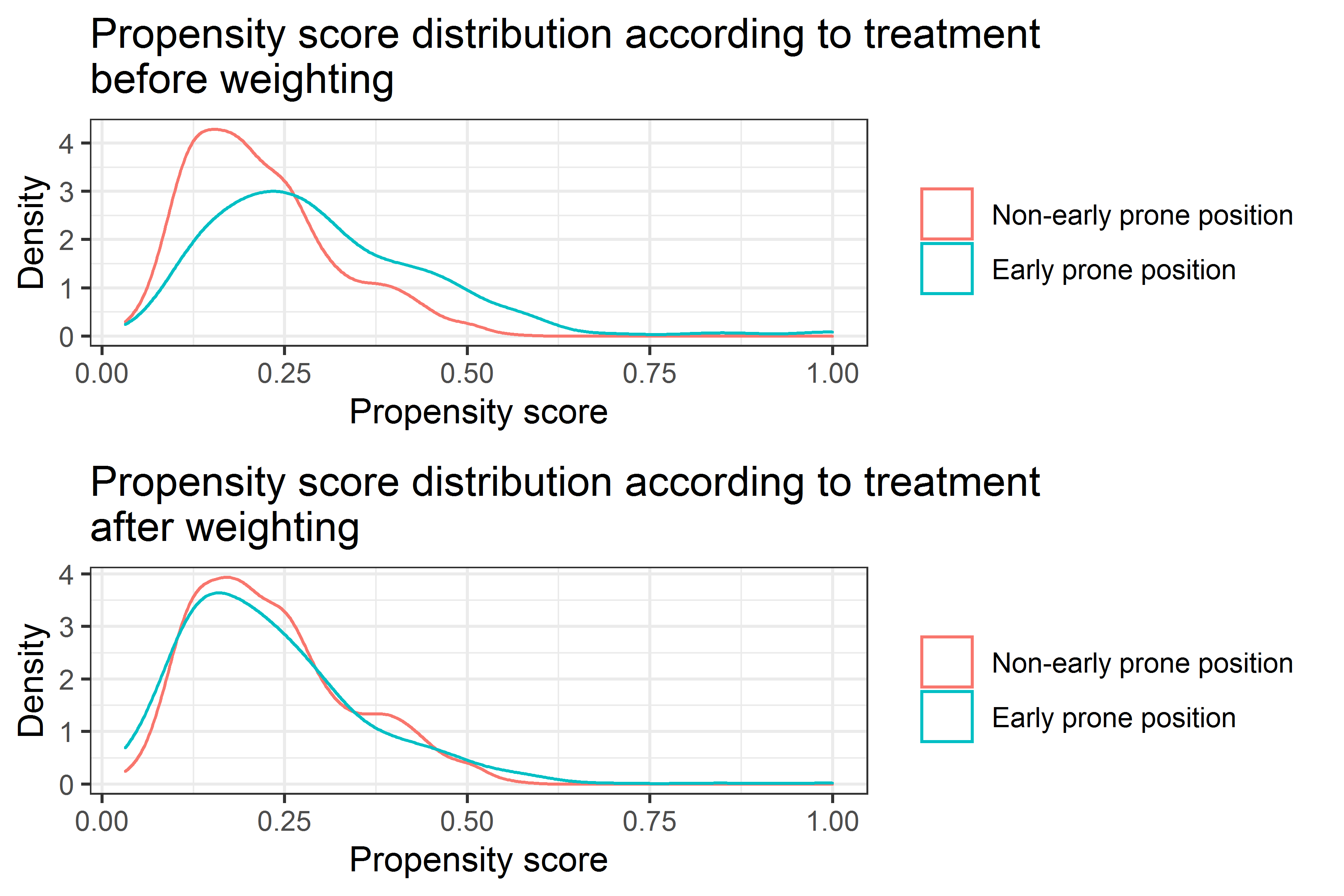
**Figure 1.** Adjustment quality before and after propensity score analysis

**Table 4**. Descriptive subgroup analysis of baseline population with P_a_O_2_/F_i_O_2_ ratio <150 mmHg at Day-1 included in propensity score analysis and complete case population

|  |  | **Baseline population (n = 1106)** | | | **Complete case population (n = 474)** | | |
| --- | --- | --- | --- | --- | --- | --- | --- |
| **label** | **N (NA)** | **Early PP (n = 310)** | **Non-early PP (n = 796)** | **p** | **Early PP (n = 329)** | **Non-early PP (n = 145)** | **p** |
| Age, years, <40 | 1106 (0) | 29 (4%) | 18 (6%) | 0.4284 | 13 (4%) | 10 (7%) | 0.3991 |
| Age, years, 40 - 59 | 1106 (0) | 280 (35%) | 111 (36%) | 0.4284 | 125 (38%) | 54 (37%) | 0.3991 |
| Age, years, 60 - 74 | 1106 (0) | 398 (50%) | 148 (48%) | 0.4284 | 165 (50%) | 66 (46%) | 0.3991 |
| Age, years, ≥ 75 | 1106 (0) | 89 (11%) | 33 (11%) | 0.4284 | 26 (8%) | 15 (10%) | 0.3991 |
| Gender, Male | 1099 (7) | 605 (76%) | 225 (73%) | 0.2837 | 268 (81%) | 106 (73%) | 0.0399 |
| Gender, Female | 1099 (7) | 187 (24%) | 82 (27%) | 0.2837 | 61 (19%) | 39 (27%) | 0.0399 |
| Frailty clinical scale, 1 - 3 | 1017 (89) | 638 (87%) | 249 (87%) | 0.8796 | 292 (89%) | 126 (87%) | 0.6487 |
| Frailty clinical scale, 4 | 1017 (89) | 64 (9%) | 25 (9%) | 0.8796 | 26 (8%) | 15 (10%) | 0.6487 |
| Frailty clinical scale, 5 - 9 | 1017 (89) | 28 (4%) | 13 (5%) | 0.8796 | 11 (3%) | 4 (3%) | 0.6487 |
| SOFA Cardiovascular system, ≥ 3 | 1088 (18) | 459 (59%) | 196 (64%) | 0.0877 | 210 (64%) | 93 (64%) | 0.9487 |
| SOFA Coagulation, ≥ 3 | 1070 (36) | 9 (1%) | 1 (0%) | 0.2997 | 3 (1%) | 0 (0%) | 0.5564 |
| SOFA Renal, ≥ 3 | 1075 (31) | 73 (9%) | 26 (9%) | 0.7337 | 32 (10%) | 11 (8%) | 0.4547 |
| Immunodepression | 1096 (10) | 50 (6%) | 14 (5%) | 0.2599 | 21 (6%) | 3 (2%) | 0.0484 |
| Treated hypertension | 1094 (12) | 395 (50%) | 153 (50%) | 0.9163 | 161 (49%) | 69 (48%) | 0.7864 |
| Diabetes | 1094 (12) | 241 (31%) | 90 (29%) | 0.6725 | 103 (31%) | 38 (26%) | 0.2630 |
| Body mass index, kg/m2, < 30 | 1023 (83) | 404 (55%) | 137 (47%) | 0.0239 | 187 (57%) | 69 (48%) | 0.1581 |
| Body mass index, kg/m2, 30 - 39 | 1023 (83) | 289 (40%) | 135 (46%) | 0.0239 | 128 (39%) | 67 (46%) | 0.1581 |
| Body mass index, kg/m2, ≥ 40 | 1023 (83) | 36 (5%) | 22 (7%) | 0.0239 | 14 (4%) | 9 (6%) | 0.1581 |
| Days between first signs and ICU admission, < 4 | 1041 (65) | 114 (15%) | 43 (14%) | 0.6345 | 53 (16%) | 14 (10%) | 0.1175 |
| Days between first signs and ICU admission, 4 - 7 | 1041 (65) | 261 (35%) | 97 (33%) | 0.6345 | 114 (35%) | 48 (33%) | 0.1175 |
| Days between first signs and ICU admission, ≥ 8 | 1041 (65) | 369 (50%) | 157 (53%) | 0.6345 | 162 (49%) | 83 (57%) | 0.1175 |
| Lymphocyte count, × 10^9/L | 922 (184) | 1 (2) | 2 (5) | 0.4086 | 1 (1) | 2 (6) | 0.0952 |
| SAPS II score | 1018 (88) | 45 (17) | 46 (17) | 0.2971 | 45 (17) | 46 (17) | 0.3606 |
| Static compliance, day 1, mL/cmH2O, < 30 | 780 (326) | 220 (41%) | 123 (52%) | 0.0123 | 134 (41%) | 78 (54%) | 0.0240 |
| Static compliance, day 1, mL/cmH2O, 30 - 39 | 780 (326) | 182 (34%) | 62 (26%) | 0.0123 | 109 (33%) | 41 (28%) | 0.0240 |
| Static compliance, day 1, mL/cmH2O, ≥ 40 | 780 (326) | 141 (26%) | 52 (22%) | 0.0123 | 86 (26%) | 26 (18%) | 0.0240 |
| Bacterial coinfection at ICU admission | 1078 (28) | 49 (6%) | 28 (9%) | 0.0905 | 23 (7%) | 16 (11%) | 0.1399 |
| ICU admission period, before March 28 | 1106 (0) | 444 (56%) | 155 (50%) | 0.0832 | 175 (53%) | 78 (54%) | 0.9037 |
| ICU admission period, after March 29 | 1106 (0) | 352 (44%) | 155 (50%) | 0.0832 | 154 (47%) | 67 (46%) | 0.9037 |

SAPS: Simplified Acute Physiology Score; SOFA: Sequential Organ Failure Assessment.

Data are mean (SD) for continuous variables and frequency (percentage) for categorical variables. N (NA): Number of observations (number of missing data). p = p value of comparison tests: Wilcoxon sum rank test for continuous variables and Chi square test or Fisher’s exact test, as appropriate, for categorical variables.

**Table 5**. Descriptive subgroup analysis of baseline population characteristics P_a_O_2_/F_i_O_2_ ratio <150 mmHg at Day-1 before and after weighted-propensity score analysis

|  | **Complete case population (n = 474)** | | | | | | **Baseline population, multiple imputation (n=1106)** | | | | | |
| --- | --- | --- | --- | --- | --- | --- | --- | --- | --- | --- | --- | --- |
|  | **Before weighting** | | | **After weighting** | | | **Before weighting** | | | **After weighting** | | |
| **label** | **Non-early PP**  **(n = 329)** | **Early PP**  **(n = 145)** | **SMD** | **Non-early PP**  **(n = 329)** | **Early PP**  **(n = 145)** | **SMD** | **Non-early PP**  **(n = 796)** | **Early PP**  **(n = 310)** | **SMD** | **Non-early PP**  **(n = 796)** | **Early PP**  **(n = 310)** | **SMD** |
| Age, years, <40 | 4% | 7% | 0.130 | 5% | 5% | 0.011 | 4% | 6% | 0.102 | 4% | 4% | 0.004 |
| Age, years, 40 - 59 | 38% | 37% | 0.015 | 38% | 38% | 0.000 | 33% | 34% | 0.019 | 33% | 32% | 0.015 |
| Age, years, 60 - 74 | 50% | 46% | 0.093 | 49% | 49% | 0.004 | 50% | 48% | 0.045 | 49% | 51% | 0.022 |
| Age, years, ≥ 75 | 8% | 10% | 0.085 | 9% | 9% | 0.000 | 14% | 13% | 0.023 | 13% | 13% | 0.011 |
| Genre, Male | 81% | 73% | 0.200 | 79% | 78% | 0.020 | 76% | 73% | 0.072 | 76% | 77% | 0.021 |
| Genre, Female | 19% | 27% | 0.200 | 21% | 22% | 0.020 | 24% | 27% | 0.072 | 24% | 23% | 0.021 |
| Frailty clinical scale, 1 - 3 | 89% | 87% | 0.057 | 88% | 87% | 0.013 | 87% | 87% | 0.020 | 87% | 87% | 0.005 |
| Frailty clinical scale, 4 | 8% | 10% | 0.085 | 9% | 8% | 0.022 | 9% | 9% | 0.010 | 9% | 9% | 0.008 |
| Frailty clinical scale, 5 - 9 | 3% | 3% | 0.034 | 3% | 4% | 0.054 | 4% | 5% | 0.026 | 4% | 4% | 0.018 |
| SOFA Cardiovascular system, ≥ 3 | 64% | 64% | 0.006 | 64% | 64% | 0.003 | 59% | 64% | 0.119 | 60% | 61% | 0.011 |
| SOFA Coagulation, ≥ 3 | 1% | 0% | 0.135 | 1% | 0% | 0.113 | 1% | 0% | 0.101 | 1% | 2% | 0.096 |
| SOFA Renal, ≥ 3 | 10% | 8% | 0.076 | 9% | 10% | 0.035 | 9% | 9% | 0.022 | 9% | 9% | 0.013 |
| Immunodepression | 6% | 2% | 0.215 | 5% | 6% | 0.041 | 6% | 5% | 0.077 | 6% | 6% | 0.011 |
| Treated hypertension | 49% | 48% | 0.027 | 48% | 45% | 0.060 | 50% | 50% | 0.010 | 50% | 51% | 0.015 |
| Diabetes | 31% | 26% | 0.113 | 30% | 29% | 0.010 | 31% | 29% | 0.030 | 30% | 31% | 0.022 |
| Body mass index, kg/m2, < 30 | 57% | 48% | 0.186 | 55% | 54% | 0.011 | 55% | 47% | 0.173 | 53% | 53% | 0.018 |
| Body mass index, kg/m2, 30 - 39 | 39% | 46% | 0.148 | 41% | 41% | 0.011 | 40% | 46% | 0.123 | 41% | 41% | 0.015 |
| Body mass index, kg/m2, ≥ 40 | 4% | 6% | 0.088 | 5% | 5% | 0.002 | 5% | 8% | 0.103 | 6% | 6% | 0.023 |
| Days between first signs and ICU admission, < 4 | 16% | 10% | 0.193 | 14% | 13% | 0.019 | 11% | 8% | 0.097 | 10% | 12% | 0.047 |
| Days between first signs and ICU admission, 4 - 7 | 35% | 33% | 0.033 | 34% | 35% | 0.020 | 30% | 31% | 0.027 | 30% | 29% | 0.019 |
| Days between first signs and ICU admission, ≥ 8 | 49% | 57% | 0.160 | 52% | 52% | 0.006 | 60% | 61% | 0.033 | 60% | 59% | 0.014 |
| Lymphocyte count, × 10^9/L | 1 (1) | 2 (6) | 0.260 | 1 (1) | 1(3) | 0.086 | 1 (2) | 2 (5) | 0.160 | 1 (3) | 1 (3) | 0.017 |
| SAPS II score | 45 (17) | 46 (17) | 0.072 | 46 (17) | 46 (17) | 0.001 | 45 (17) | 46 (17) | 0.057 | 46 (17) | 46 (17) | 0.053 |
| Static compliance, day 1, mL/cmH2O, < 30 | 41% | 54% | 0.263 | 45% | 45% | 0.000 | 43% | 49% | 0.137 | 45% | 44% | 0.013 |
| Static compliance, day 1, mL/cmH2O, 30 - 39 | 33% | 28% | 0.105 | 32% | 30% | 0.043 | 32% | 27% | 0.106 | 31% | 30% | 0.021 |
| Static compliance, day 1, mL/cmH2O, ≥ 40 | 26% | 18% | 0.199 | 24% | 26% | 0.045 | 25% | 23% | 0.047 | 25% | 26% | 0.037 |
| Bacterial coinfection at ICU admission | 7% | 11% | 0.141 | 8% | 8% | 0.006 | 6% | 10% | 0.117 | 7% | 7% | 0.006 |
| ICU admission period, before March 28 | 53% | 54% | 0.012 | 53% | 54% | 0.024 | 56% | 50% | 0.116 | 54% | 53% | 0.017 |
| ICU admission period, after March 29 | 47% | 46% | 0.012 | 47% | 46% | 0.024 | 44% | 50% | 0.116 | 46% | 47% | 0.017 |

SAPS: Simplified Acute Physiology Score; SOFA: Sequential Organ Failure Assessment.

Data are mean (SD) for continuous variables and percentages for categorical variables; SMD: Standardized Mean difference; After weighting: mean, SD and percentages are weighted by IPTW (Inverse of Probability of Treatment Weighted)


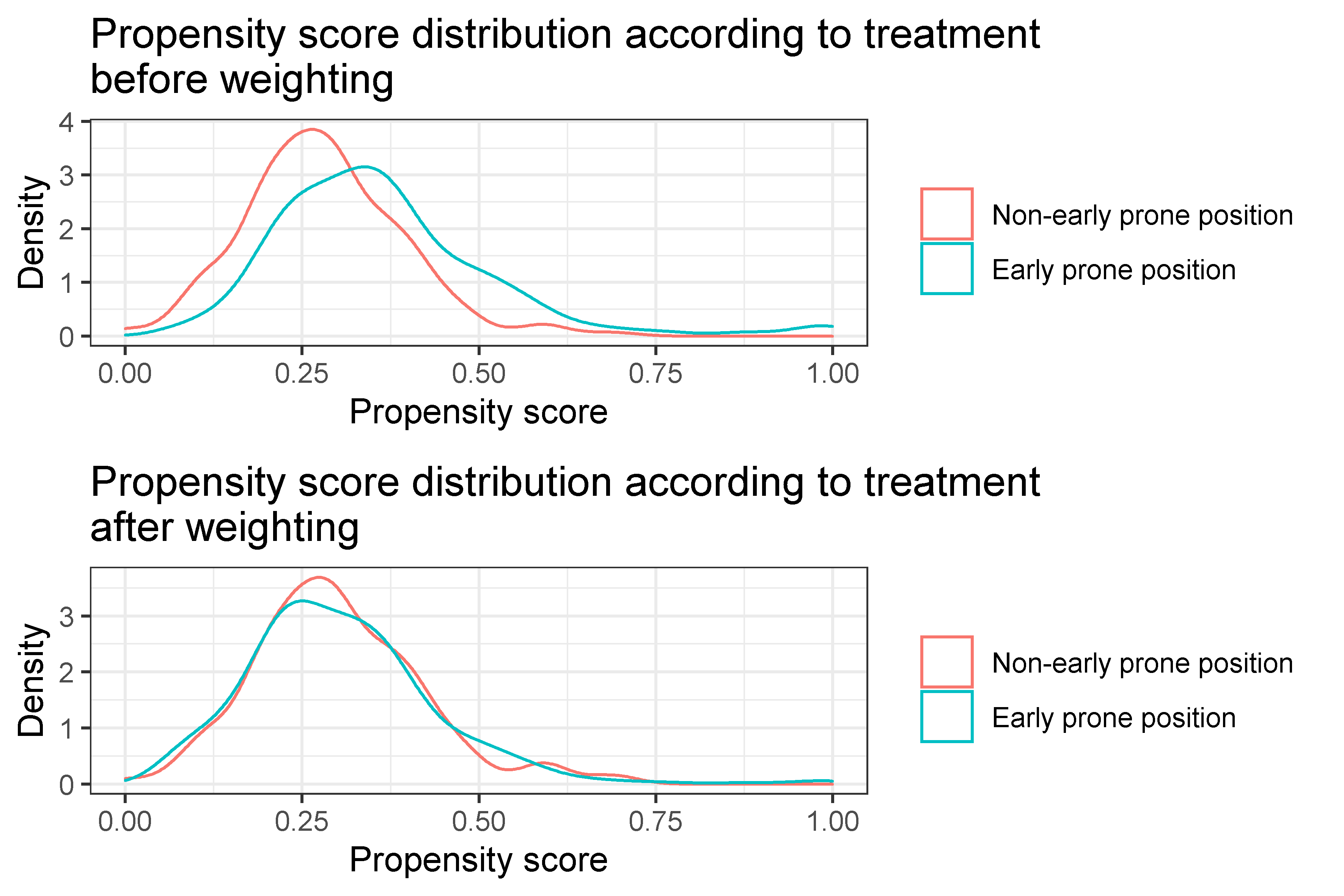
**Figure 2.** Adjustment quality before and after propensity score analysis in the subgroup of patients P_a_O_2_/F_i_O_2_ ratio <150 mmHg at Day-1

**Figure 3.** **a.** Kaplan Meier curves according to prone status in ICU at Day-1 before weighting adjustment in complete case subgroup population with P_a_O_2_/F_i_O_2_ ratio <150 mmHg. **b.** Kaplan Meier curves according to prone status in ICU at Day-1 after weighting adjustment in complete case subgroup population with P_a_O_2_/F_i_O_2_ ratio <150 mmHg. ICU: intensive care unit

a

b


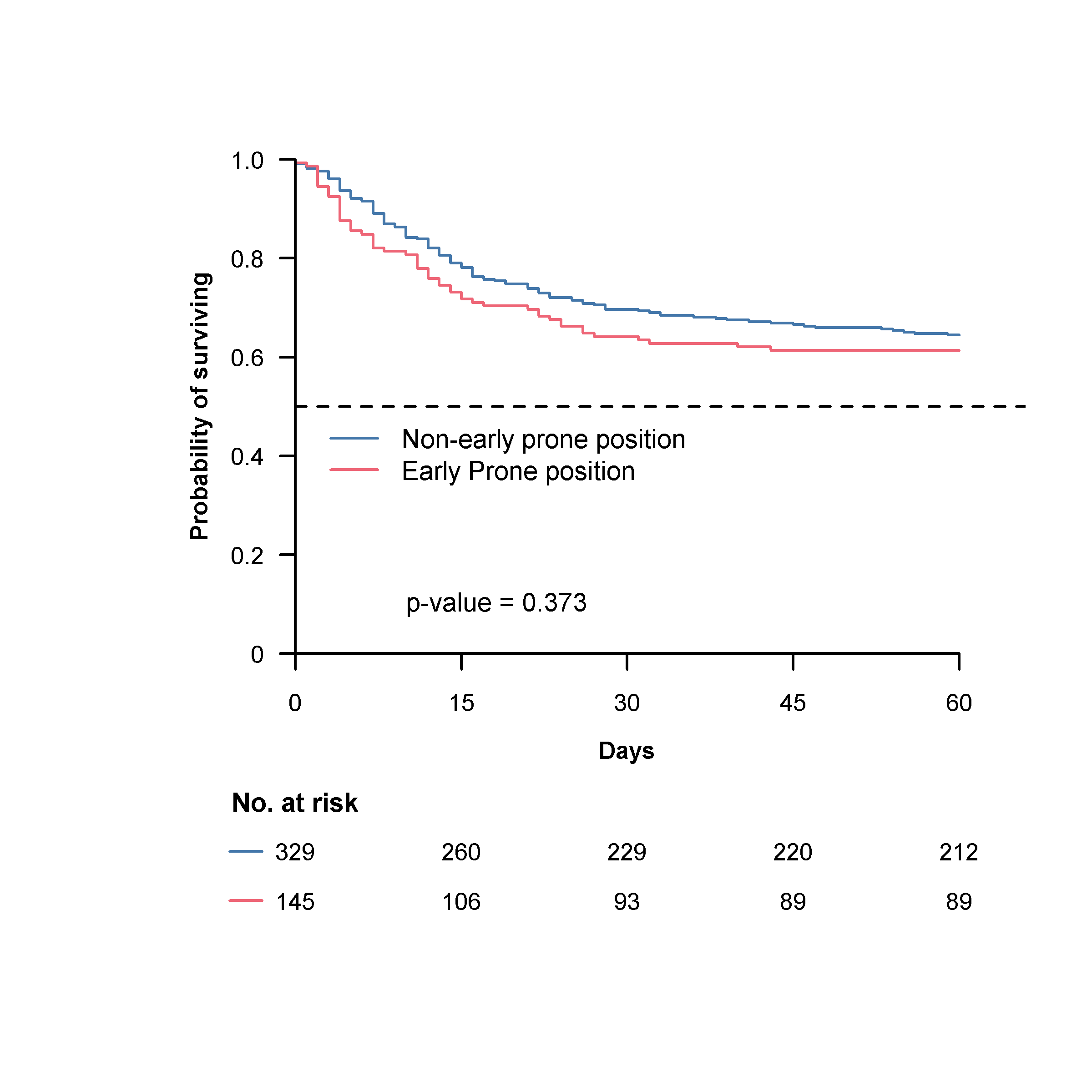

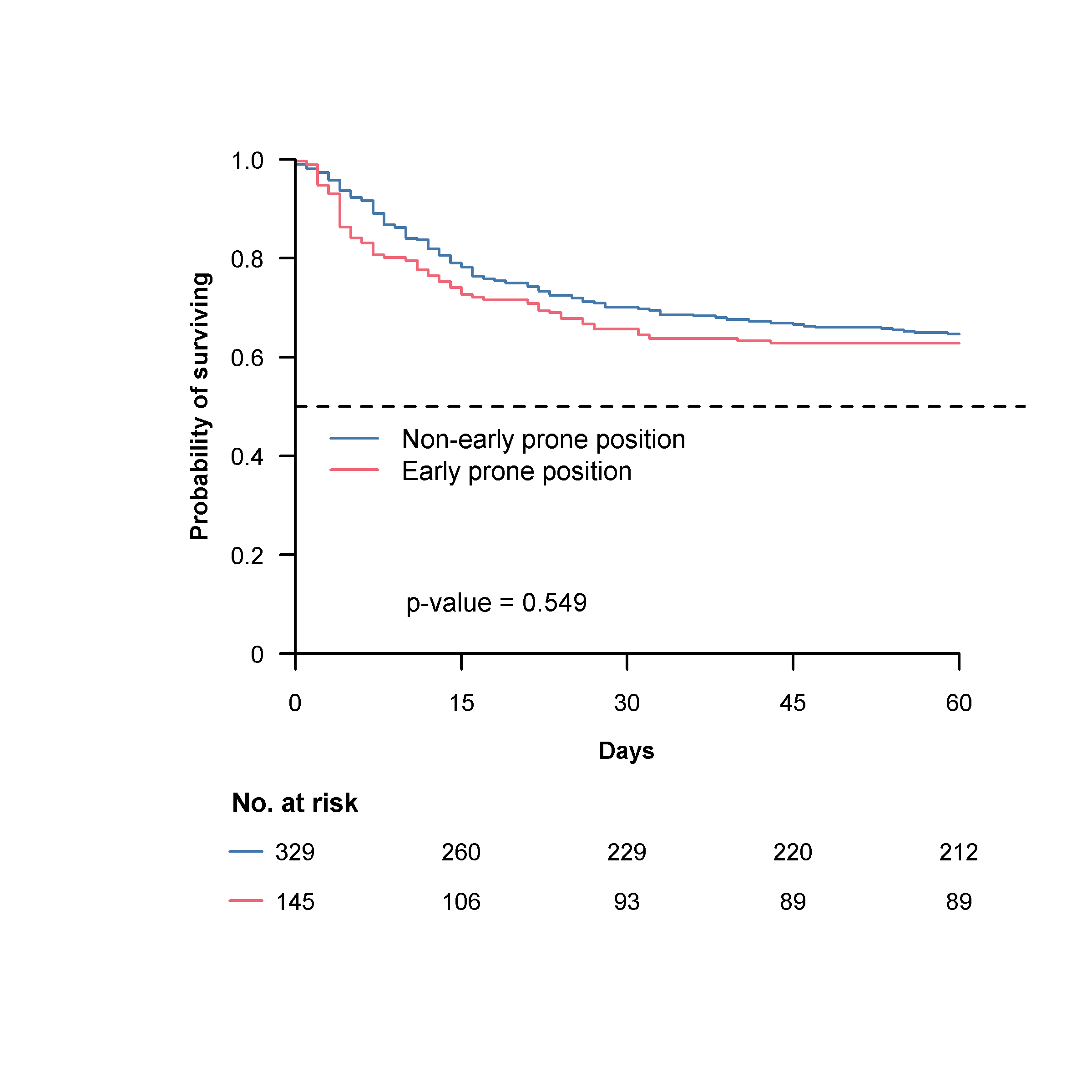


**Figure 4.** **a.** Forest plot: Hazard Ratio according to prone status in ICU at Day-1 before and after weighting in complete case subgroup population with P_a_O_2_/F_i_O_2_ ratio <150 mmHg. **b.** Hazard Ratio according to prone status in ICU at Day-1 before and after weighting in baseline subgroup population with P_a_O_2_/F_i_O_2_ ratio <150 mmHg. ICU: intensive care unit; HR: Hazard ratio.


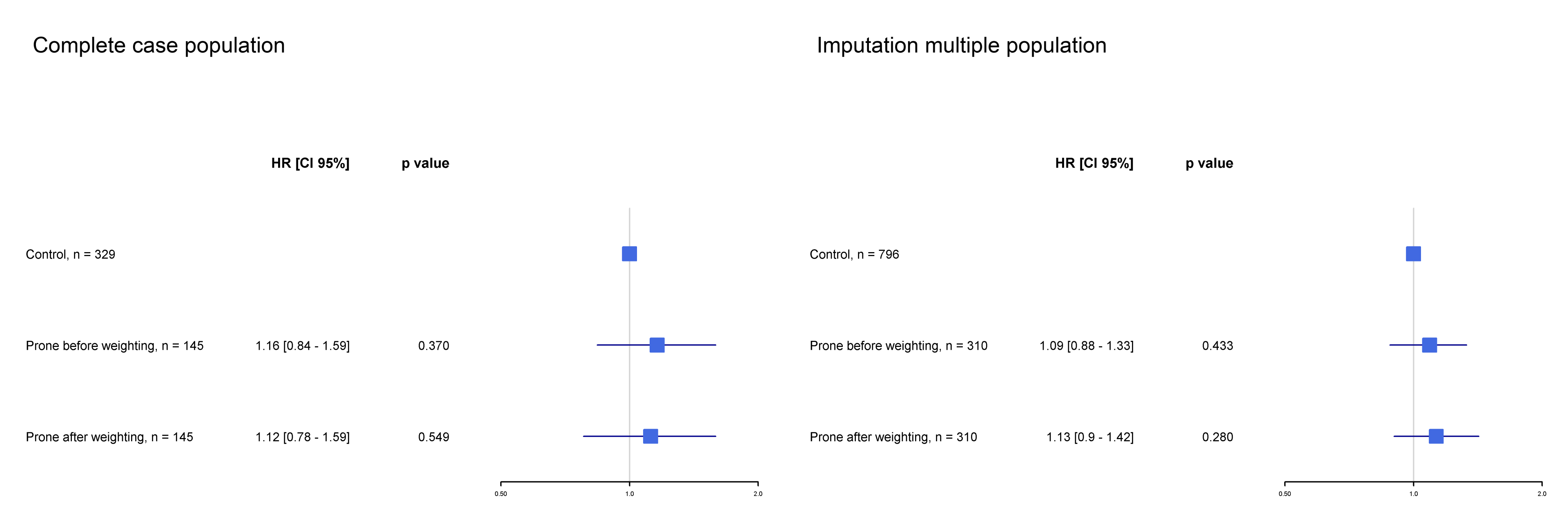


**Table 6**. Descriptive subgroup analysis of baseline population with P_a_O_2_/F_i_O_2_ ratio >150 mmHg included in propensity score analysis and complete case population

|  |  | **Baseline population (n = 1031)** | | | **Complete case population (n = 470)** | | |
| --- | --- | --- | --- | --- | --- | --- | --- |
| **label** | **N (NA)** | **Early PP**  **(n = 181)** | **Non-early PP**  **(n = 850)** | **p** | **Early PP**  **(n = 78)** | **Non-early PP**  **(n = 392)** | **p** |
| Age, years, <40 | 1031 (0) | 34 (4%) | 7 (4%) | 0.9747 | 18 (5%) | 4 (5%) | 0.9370 |
| Age, years, 40 - 59 | 1031 (0) | 299 (35%) | 65 (36%) | 0.9747 | 138 (35%) | 25 (32%) | 0.9370 |
| Age, years, 60 - 74 | 1031 (0) | 408 (48%) | 88 (49%) | 0.9747 | 189 (48%) | 40 (51%) | 0.9370 |
| Age, years, ≥ 75 | 1031 (0) | 109 (13%) | 21 (12%) | 0.9747 | 47 (12%) | 9 (12%) | 0.9370 |
| Genre, Male | 1028 (3) | 637 (75%) | 131 (73%) | 0.5118 | 294 (75%) | 58 (74%) | 0.9051 |
| Genre, Female | 1028 (3) | 211 (25%) | 49 (27%) | 0.5118 | 98 (25%) | 20 (26%) | 0.9051 |
| Frailty clinical scale, 1 - 3 | 918 (113) | 648 (86%) | 149 (91%) | 0.0326 | 338 (86%) | 72 (92%) | 0.1419 |
| Frailty clinical scale, 4 | 918 (113) | 69 (9%) | 13 (8%) | 0.0326 | 37 (9%) | 6 (8%) | 0.1419 |
| Frailty clinical scale, 5 - 9 | 918 (113) | 38 (5%) | 1 (1%) | 0.0326 | 17 (4%) | 0 (0%) | 0.1419 |
| SOFA Cardiovascular system, ≥ 3 | 1020 (11) | 548 (65%) | 109 (62%) | 0.4500 | 276 (70%) | 52 (67%) | 0.5110 |
| SOFA Coagulation, ≥ 3 | 1002 (29) | 5 (1%) | 2 (1%) | 0.3415 | 2 (1%) | 1 (1%) | 0.4206 |
| SOFA Renal, ≥ 3 | 1005 (26) | 64 (8%) | 13 (8%) | 0.9362 | 30 (8%) | 8 (10%) | 0.4411 |
| Immunodepression | 1021 (10) | 70 (8%) | 20 (11%) | 0.2204 | 36 (9%) | 8 (10%) | 0.7664 |
| Treated hypertension | 1019 (12) | 391 (47%) | 116 (64%) | <0.0001 | 177 (45%) | 56 (72%) | <0.0001 |
| Diabetes | 1021 (10) | 205 (24%) | 65 (36%) | 0.0010 | 99 (25%) | 28 (36%) | 0.0532 |
| Body mass index, kg/m2, < 30 | 975 (56) | 498 (62%) | 71 (43%) | <0.0001 | 238 (61%) | 36 (46%) | 0.0310 |
| Body mass index, kg/m2, 30 - 39 | 975 (56) | 280 (35%) | 78 (47%) | <0.0001 | 139 (35%) | 36 (46%) | 0.0310 |
| Body mass index, kg/m2, ≥ 40 | 975 (56) | 31 (4%) | 17 (10%) | <0.0001 | 15 (4%) | 6 (8%) | 0.0310 |
| Days between first signs and ICU admission, < 4 | 964 (67) | 86 (11%) | 20 (12%) | 0.3567 | 48 (12%) | 13 (17%) | 0.5353 |
| Days between first signs and ICU admission, 4 - 7 | 964 (67) | 280 (35%) | 68 (40%) | 0.3567 | 140 (36%) | 28 (36%) | 0.5353 |
| Days between first signs and ICU admission, ≥ 8 | 964 (67) | 429 (54%) | 81 (48%) | 0.3567 | 204 (52%) | 37 (47%) | 0.5353 |
| Lymphocyte count, × 10^9/L | 895 (136) | 1 (2) | 2 (9) | 0.5692 | 1 (1) | 2 (11) | 0.9726 |
| SAPS II score | 962 (69) | 45 (17) | 43 (15) | 0.4233 | 46 (17) | 44 (15) | 0.6149 |
| Static compliance, day 1, mL/cmH2O, < 30 | 719 (312) | 170 (29%) | 45 (34%) | 0.3592 | 111 (28%) | 25 (32%) | 0.7891 |
| Static compliance, day 1, mL/cmH2O, 30 - 39 | 719 (312) | 205 (35%) | 46 (35%) | 0.3592 | 134 (34%) | 26 (33%) | 0.7891 |
| Static compliance, day 1, mL/cmH2O, ≥ 40 | 719 (312) | 213 (36%) | 40 (31%) | 0.3592 | 147 (38%) | 27 (35%) | 0.7891 |
| Bacterial coinfection at ICU admission | 999 (32) | 44 (5%) | 9 (5%) | 0.8400 | 17 (4%) | 2 (3%) | 0.7524 |
| ICU admission period, before March 28 | 1031 (0) | 492 (58%) | 101 (56%) | 0.6070 | 239 (61%) | 48 (62%) | 0.9250 |
| ICU admission period, after March 29 | 1031 (0) | 358 (42%) | 80 (44%) | 0.6070 | 153 (39%) | 30 (38%) | 0.9250 |

SAPS: Simplified Acute Physiology Score; SOFA: Sequential Organ Failure Assessment.

Data are mean (SD) for continuous variables and frequency (percentage) for categorical variables. N (NA): Number of observations (number of missing data).

p = p value of comparison tests: Wilcoxon sum rank test for continuous variables and Chi square test or Fisher’s exact test, as appropriate, for categorical variables.

**Table 7**. Descriptive subgroup analysis of baseline population characteristics with P_a_O_2_/F_i_O_2_ ratio >150 mmHg at Day-1 before and after weighted-propensity score analysis

|  | **Complete case population (n = 470)** | | | | | | **Baseline population, multiple imputation (n = 1031)** | | | | | |
| --- | --- | --- | --- | --- | --- | --- | --- | --- | --- | --- | --- | --- |
|  | **Before weighting** | | | **After weighting** | | | **Before weighting** | | | **After weighting** | | |
| **label** | **Non-early PP**  **(n = 392)** | **Early PP**  **(n = 78)** | **SMD** | **Non-early PP**  **(n = 392)** | **Early PP**  **(n = 78)** | **SMD** | **Non-early PP**  **(n = 850)** | **Early PP**  **(n = 181)** | **SMD** | **Non-early PP**  **(n = 850)** | **Early PP**  **(n = 381)** | **SMD** |
| Age, years, <40 | 5% | 5% | 0.025 | 5% | 4% | 0.055 | 4% | 4% | 0.007 | 4% | 3% | 0.054 |
| Age, years, 40 - 59 | 35% | 32% | 0.067 | 35% | 37% | 0.046 | 32% | 34% | 0.036 | 32% | 31% | 0.035 |
| Age, years, 60 - 74 | 48% | 51% | 0.061 | 49% | 52% | 0.053 | 48% | 49% | 0.012 | 48% | 52% | 0.081 |
| Age, years, ≥ 75 | 12% | 12% | 0.014 | 12% | 8% | 0.127 | 16% | 14% | 0.061 | 16% | 14% | 0.041 |
| Genre, Male | 75% | 74% | 0.015 | 75% | 76% | 0.033 | 75% | 73% | 0.055 | 74% | 76% | 0.050 |
| Genre, Female | 25% | 26% | 0.015 | 25% | 24% | 0.033 | 25% | 27% | 0.055 | 26% | 24% | 0.050 |
| Frailty clinical scale, 1 - 3 | 86% | 92% | 0.197 | 87% | 94% | 0.229 | 86% | 92% | 0.184 | 87% | 87% | 0.038 |
| Frailty clinical scale, 4 | 9% | 8% | 0.062 | 9% | 6% | 0.114 | 9% | 8% | 0.050 | 9% | 7% | 0.071 |
| Frailty clinical scale, 5 - 9 | 4% | 0% | 0.301 | 4% | 0% | 0.274 | 5% | 1% | 0.260 | 4% | 7% | 0.092 |
| SOFA Cardiovascular system, ≥ 3 | 70% | 67% | 0.080 | 70% | 72% | 0.039 | 65% | 62% | 0.055 | 65% | 68% | 0.071 |
| SOFA Coagulation, ≥ 3 | 1% | 1% | 0.082 | 1% | 1% | 0.017 | 1% | 1% | 0.055 | 1% | 1% | 0.022 |
| SOFA Renal, ≥ 3 | 8% | 10% | 0.091 | 8% | 7% | 0.034 | 8% | 7% | 0.013 | 8% | 7% | 0.023 |
| Immunodepression | 9% | 10% | 0.036 | 9% | 9% | 0.022 | 8% | 11% | 0.093 | 9% | 8% | 0.018 |
| Treated hypertension | 45% | 72% | 0.560 | 50% | 47% | 0.047 | 47% | 64% | 0.363 | 50% | 54% | 0.089 |
| Diabetes | 25% | 36% | 0.232 | 27% | 25% | 0.043 | 24% | 36% | 0.260 | 26% | 24% | 0.048 |
| Body mass index, kg/m2, < 30 | 61% | 46% | 0.294 | 58% | 58% | 0.012 | 62% | 43% | 0.378 | 58% | 59% | 0.032 |
| Body mass index, kg/m2, 30 - 39 | 35% | 46% | 0.218 | 37% | 37% | 0.006 | 35% | 47% | 0.255 | 37% | 36% | 0.029 |
| Body mass index, kg/m2, ≥ 40 | 4% | 8% | 0.166 | 5% | 6% | 0.041 | 4% | 10% | 0.243 | 5% | 5% | 0.012 |
| Days between first signs and ICU admission, < 4 | 12% | 17% | 0.125 | 13% | 13% | 0.006 | 7% | 8% | 0.017 | 7% | 7% | 0.030 |
| Days between first signs and ICU admission, 4 - 7 | 36% | 36% | 0.004 | 36% | 34% | 0.042 | 30% | 36% | 0.136 | 31% | 29% | 0.038 |
| Days between first signs and ICU admission, ≥ 8 | 52% | 47% | 0.092 | 51% | 54% | 0.044 | 63% | 56% | 0.132 | 62% | 64% | 0.050 |
| Lymphocyte count, × 10^9/L | 1 (1) | 2 (11) | 0.174 | 1 (1) | 1 (5) | 0.012 | 1 (2) | 2 (10) | 0.156 | 1 (2) | 1 (5) | 0.018 |
| SAPS II score | 46 (17) | 44 (15) | 0.096 | 45 (17) | 44 (16) | 0.096 | 45 (17) | 43 (15) | 0.121 | 45 (17) | 45 (17) | 0.027 |
| Static compliance, day 1, mL/cmH2O, < 30 | 28% | 32% | 0.081 | 30% | 41% | 0.243 | 29% | 36% | 0.158 | 31% | 32% | 0.034 |
| Static compliance, day 1, mL/cmH2O, 30 - 39 | 34% | 33% | 0.018 | 34% | 31% | 0.063 | 34% | 35% | 0.032 | 35% | 35% | 0.049 |
| Static compliance, day 1, mL/cmH2O, ≥ 40 | 38% | 35% | 0.060 | 37% | 28% | 0.184 | 37% | 28% | 0.180 | 35% | 34% | 0.064 |
| Bacterial coinfection at ICU admission | 4% | 3% | 0.097 | 4% | 7% | 0.114 | 6% | 5% | 0.024 | 5% | 6% | 0.036 |
| ICU admission period, before March 28 | 61% | 62% | 0.012 | 61% | 56% | 0.103 | 58% | 56% | 0.042 | 57% | 52% | 0.100 |
| ICU admission period, after March 29 | 39% | 38% | 0.012 | 39% | 44% | 0.103 | 42% | 44% | 0.042 | 43% | 48% | 0.100 |

SAPS: Simplified Acute Physiology Score; SOFA: Sequential Organ Failure Assessment.

Data are mean (SD) for continuous variables and percentages for categorical variables; SMD: Standardized Mean difference; After weighting: mean, SD and percentages are weighted by IPTW (Inverse of Probability of Treatment Weighted).

**Figure 5.** Adjustment quality before and after propensity score analysis in the subgroup of patients with P_a_O_2_/F_i_O_2_ ratio >150 mmHg at Day-1


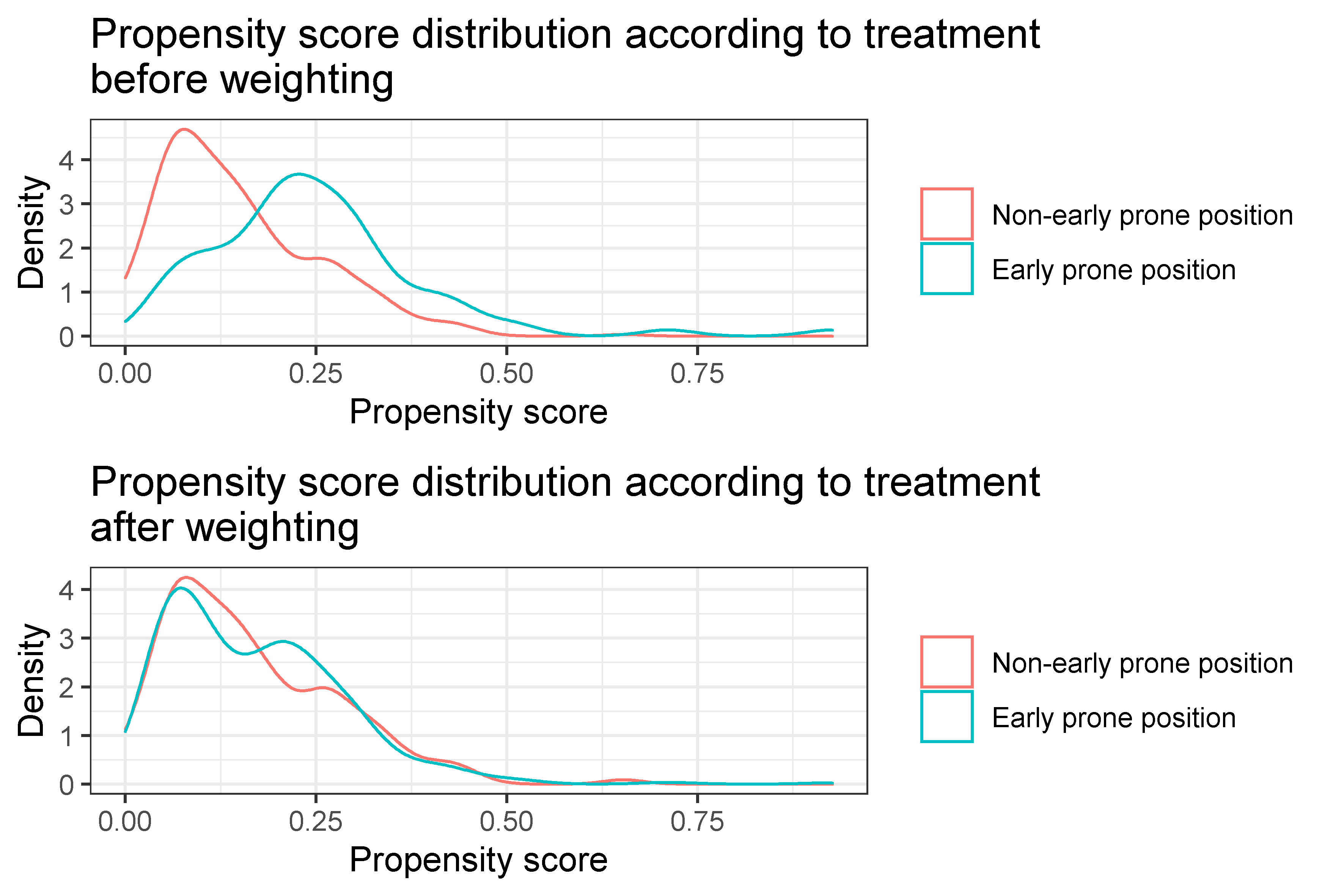


## Figure 6. a. Kaplan Meier curves according to prone status in ICU at Day-1 before weighting adjustment in complete case subgroup population with P_a_O_2_/F_i_O_2_ ratio >150 mmHg. b. Kaplan Meier curves according to prone status in ICU at Day-1 after weighting adjustment in complete case subgroup population with P_a_O_2_/F_i_O_2_ ratio >150 mmHg. ICU: intensive care unit.

a

b


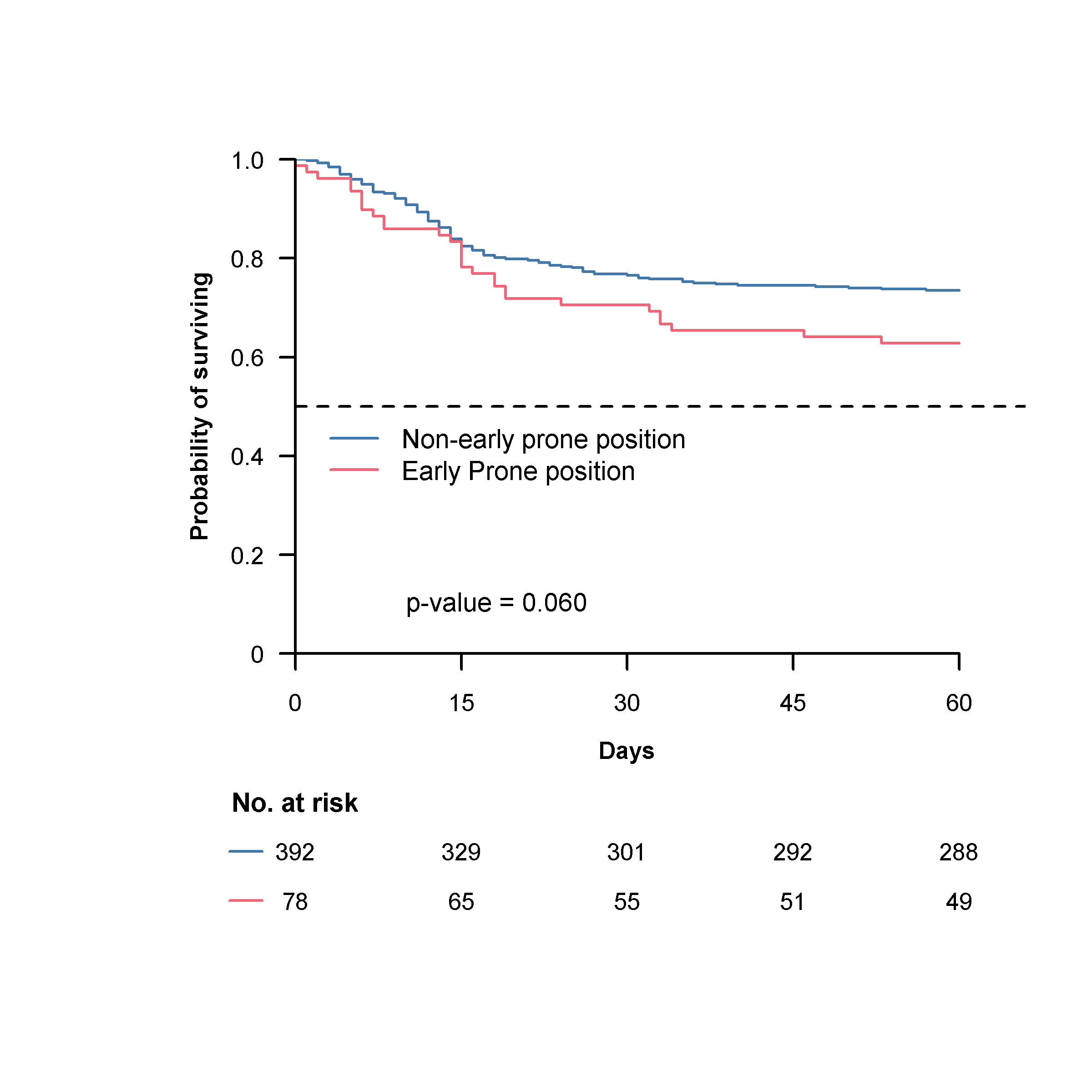

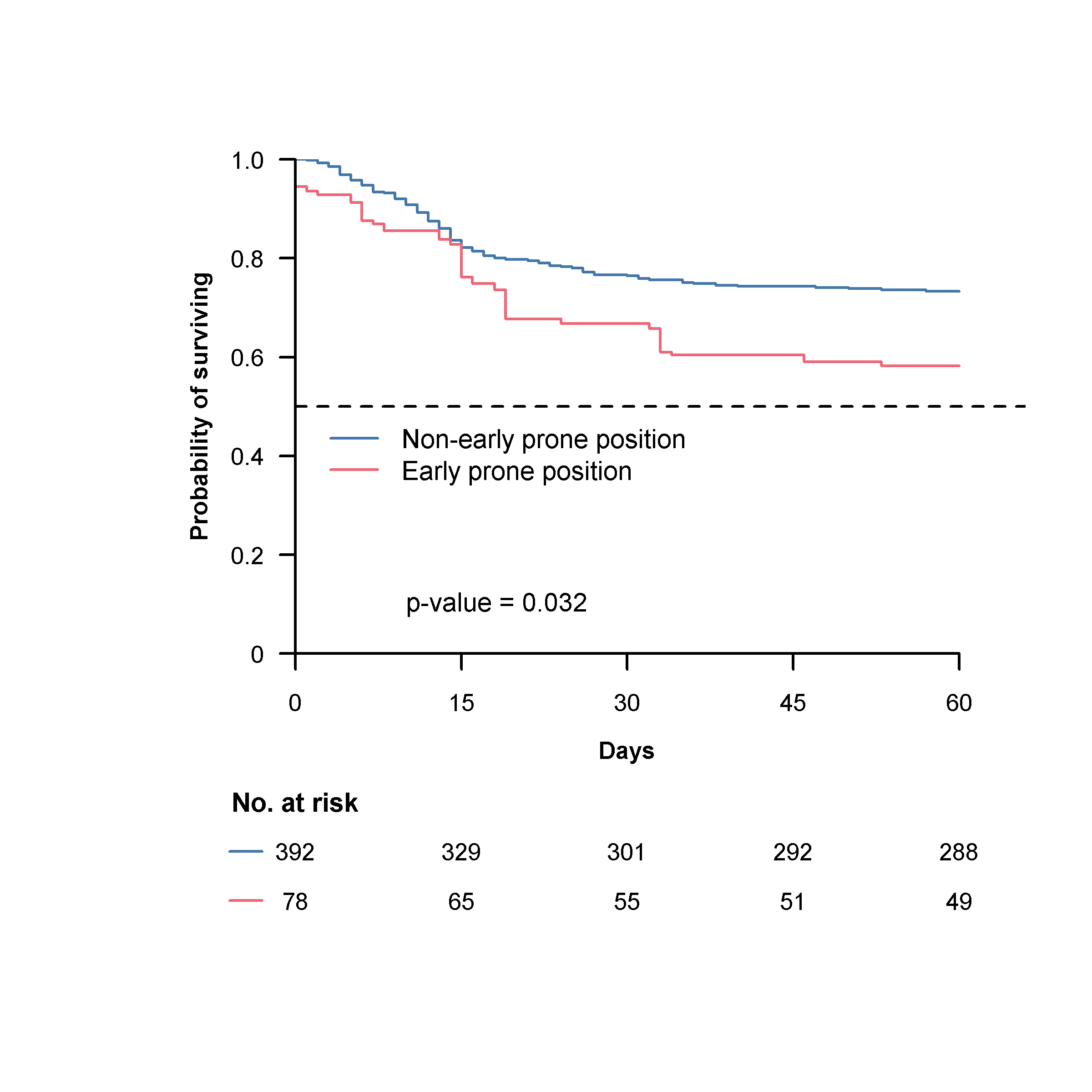


**Figure 7. a.** Forest plot: Hazard Ratio according to prone status in ICU at Day-1 before and after weighting in complete case subgroup population with P_a_O_2_/F_i_O_2_ ratio >150 mmHg. **b.** Hazard Ratio according to prone status in ICU at Day-1 before and after weighting in baseline subgroup population with P_a_O_2_/F_i_O_2_ ratio >150 mmHg. ICU: intensive care unit; HR: Hazard ratio.


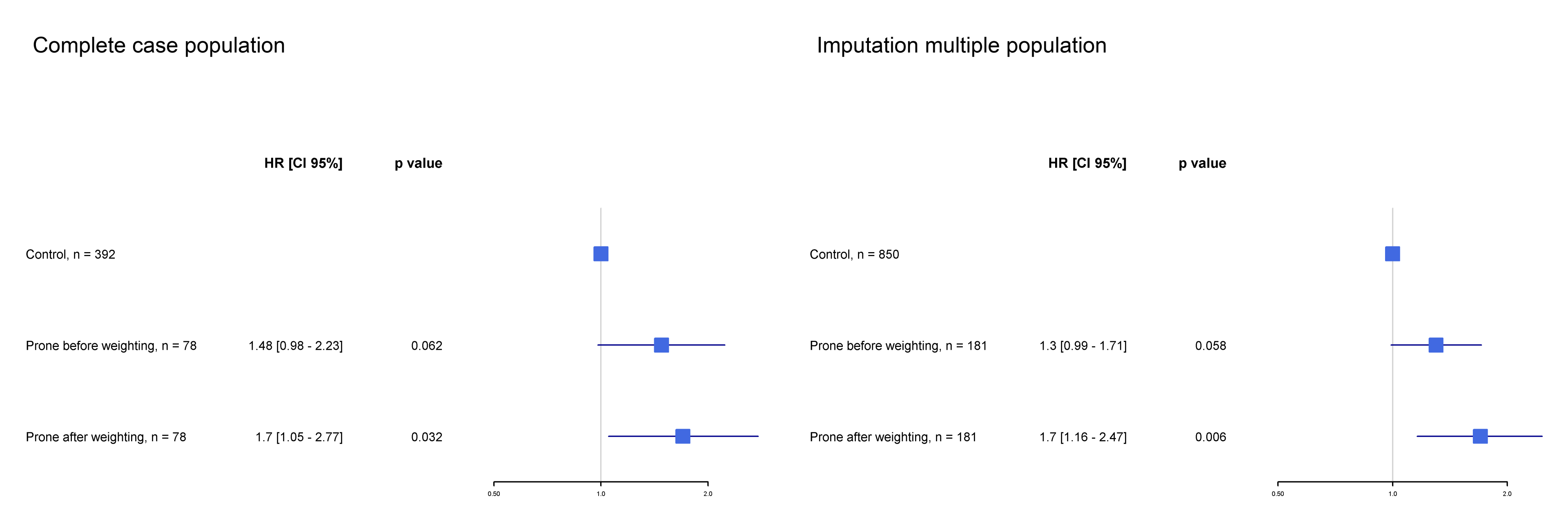

Supplement: Supplementary file 1 — Additional file 1. Additional information about the baseline characteristics and the statistical analysis (file format in .docx). Additional Tables and Figures. Table S1. Distribution of patients per region included in this study according to their prone position status at Day-1. Table S2. Descriptive analysis of baseline population included in propensity score analysis and complete case population. Table S3. Descriptive analysis of baseline characteristics before and after weighted-propensity score analysis. Fig. S1. Adjustment quality before and after propensity score analysis. Table S4. Descriptive subgroup analysis of baseline population with PaO2/FiO2 ratio < 150 mmHg at Day-1 included in propensity score analysis and complete case population. Table S5. Descriptive subgroup analysis of baseline population characteristics with PaO2/FiO2 ratio < 150 mmHg at Day-1 before and after weighted-propensity score analysis. Fig. S2. Adjustment quality before and after propensity score analysis in the subgroup of patients with PaO2/FiO2 ratio < 150 mmHg at Day-1. Fig. S3. a Kaplan–Meier curves according to prone status in ICU at Day-1 before weighting adjustment in complete case subgroup population with PaO2/FiO2 ratio < 150 mmHg. b Kaplan–Meier curves according to prone status in ICU at Day-1 after weighting adjustment in complete case subgroup population with PaO2/FiO2 < 150 mmHg. Fig. S4. a Forest plot: Hazard Ratio according to prone status in ICU at Day-1 before and after weighting in complete case subgroup population with PaO2/FiO2 ratio < 150 mmHg. b Hazard Ratio according to prone status in ICU at Day-1 before and after weighting in baseline subgroup population with PaO2/FiO2 ratio < 150 mmHg. Table S6. Descriptive subgroup analysis of baseline population with PaO2/FiO2 ratio > 150 mmHg included in propensity score analysis and complete case population. Table S7. Descriptive subgroup analysis of baseline population characteristics with PaO2/FiO2 ratio > 150 mmHg at [file 13054_2022_3949_MOESM1_ESM.docx]
